# Conformational Conversion during Amyloid Formation at Atomic Resolution

**DOI:** 10.1016/j.molcel.2010.11.028

**Published:** 2011-01-21

**Authors:** Timo Eichner, Arnout P. Kalverda, Gary S. Thompson, Steve W. Homans, Sheena E. Radford

**Affiliations:** 1 Astbury Centre for Structural Molecular Biology and Institute of Molecular and Cellular Biology, University of Leeds, Leeds LS2 9JT, UK

## Abstract

Numerous studies of amyloid assembly have indicated that partially folded protein species are responsible for initiating aggregation. Despite their importance, the structural and dynamic features of amyloidogenic intermediates and the molecular details of how they cause aggregation remain elusive. Here, we use ΔN6, a truncation variant of the naturally amyloidogenic protein β_2_-microglobulin (β_2_m), to determine the solution structure of a nonnative amyloidogenic intermediate at high resolution. The structure of ΔN6 reveals a major repacking of the hydrophobic core to accommodate the nonnative peptidyl-prolyl *trans*-isomer at Pro32. These structural changes, together with a concomitant pH-dependent enhancement in backbone dynamics on a microsecond-millisecond timescale, give rise to a rare conformer with increased amyloidogenic potential. We further reveal that catalytic amounts of ΔN6 are competent to convert nonamyloidogenic human wild-type β_2_m (Hβ_2_m) into a rare amyloidogenic conformation and provide structural evidence for the mechanism by which this conformational conversion occurs.

## Graphical abstract

**Graphical Abstract:**

**Figure:**
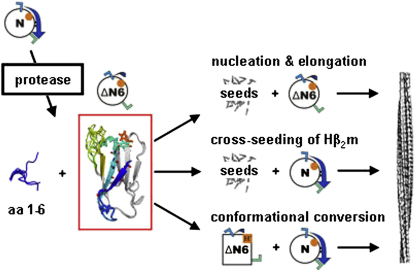

**Highlights:**

► High-resolution structure of a nonnative, amyloidogenic intermediate ► Protein dynamics reveal rare species involved in amyloid formation ► Mechanism of pH-induced enhancement of amyloidogenicity ► NMR reveals a mechanism of conformational conversion in atomistic detail

## Introduction

A number of human diseases involve protein misfolding events that ultimately result in the malfunctioning of the cellular machinery (Welch, 2004; Gidalevitz et al., 2006). In one class of these disorders, normally soluble proteins self-associate to form fibrillar aggregates known as amyloid (Westermark et al., 2007). Studies have suggested that equilibration between a natively folded protein and one or more partially or more highly unfolded species is a key initiating event in amyloid formation (Booth et al., 1997; Calamai et al., 2005). In particular, for transmissible spongiform encephalopathies, the protein-only hypothesis describes the potential of infectious nonnative protein conformations (PrP^SC^) to transmit their biophysical properties onto native protein conformers (PrP^C^), leading to the propagation of protein misfolding and aggregation (Sindi and Serio, 2009). Studies on Alzheimer's disease, Parkinson's disease, and systemic and senile amyloidosis (Kane et al., 2000; Lundmark et al., 2002; Rocken and Shakespeare, 2002; Xing et al., 2002; Angot and Brundin, 2009) have suggested that prion-like behavior may be a general feature of misfolded proteins (Brundin et al., 2010). However, the molecular mechanism of conformational conversion remains elusive.

Crucial to the understanding of the early stages of amyloid assembly is the elucidation of the structural changes that occur when a normally soluble protein becomes aggregation prone. Structural investigation of early aggregation-prone species is hampered, however, by their transient nature, heterogeneity, and instability (Calamai et al., 2005; Jahn and Radford, 2008). In general, the creation of unsatisfied hydrogen bond donors and acceptors, increased hydrophobic surface area, and/or loss of so-called negative design features (Richardson and Richardson, 2002) has been implicated in increasing amyloid potential (Liu et al., 2000; Monti et al., 2005; Ahn et al., 2006; Qin et al., 2007; Calabrese et al., 2008). However, the structural details of such changes and precisely how they engender amyloidogenicity remain unclear.

Here, we utilized β_2_-microglobulin (β_2_m), a 99-residue protein with an immunoglobulin fold (Becker and Reeke, 1985), to investigate the initiating events of amyloid assembly in all-atom detail. β_2_m is the major component of fibrillar deposits in patients with dialysis-related amyloidosis (DRA) (Gejyo et al., 1985). While the concentration of monomeric β_2_m is a risk factor for amyloid deposition in DRA, the native monomer is not able to assemble into amyloid fibrils spontaneously at neutral pH in the absence of additional factors (reviewed in Calabrese and Miranker, 2009; Platt and Radford, 2009). Instead, an increase in concentration of the nonnative, amyloidogenic precursor I_T_, a monomeric β_2_m conformer that contains a nonnative peptidyl-prolyl *trans*-isomer of Pro32, has been shown to be a key trigger of amyloid formation (Chiti et al., 2001; Jahn et al., 2006; Eichner and Radford, 2009). Furthermore, the presence of a *trans*-prolyl peptide bond at residue 32 (rather than *trans*-Ala32, Val32, or Gly32 [Eakin et al., 2006; Jahn et al., 2006; Sakata et al., 2008]) is required to form a species able to nucleate amyloid formation, presumably because of conformational restrictions imposed by the X-Pro32 *trans*-peptide bond itself (Eichner and Radford, 2009). The detailed structural changes occurring during interconversion between native β_2_m and the I_T_ state, however, remain elusive, despite a number of studies using NMR (Jahn et al., 2006; Mimmi et al., 2006; Kameda et al., 2009; Corazza et al., 2010) and X-ray crystallography (Eakin et al., 2006; Calabrese et al., 2008).

Here, we used ΔN6, a truncation variant of human β_2_m found in amyloid deposits of patients with DRA that lacks the N-terminal six amino acids and closely mimics I_T_ (Eichner and Radford, 2009), to determine the solution structure of this nonnative, amyloidogenic intermediate at high resolution using NMR spectroscopy. The results reveal a remarkable repacking of the hydrophobic core that is reminiscent of, but distinct from, the nonamyloidogenic Cu^2+^-bound hexameric state previously captured by crystallography (Calabrese et al., 2008). Most strikingly, we show that ΔN6 is able to interact specifically with human wild-type β_2_m (Hβ_2_m), causing it to adopt an amyloid-competent structure and thereby reveal the mechanism of conformational conversion of a naturally occurring amyloidogenic protein in atomistic detail.

## Results

### Real-Time NMR Studies Confirm the Structural Resemblance of ΔN6 and I_T_

**Figure 1:**
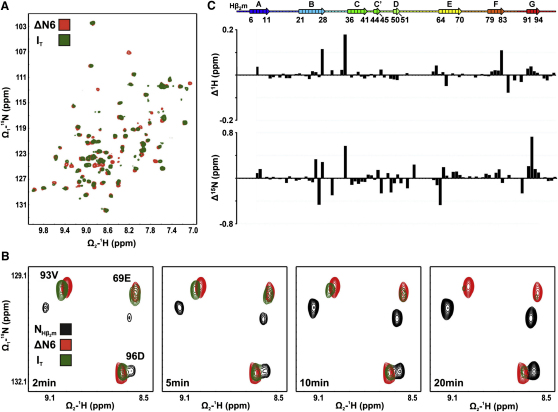
^1^H-^15^N SOFAST HMQC Spectra and Chemical Shift Analysis of ΔN6 and I_T_ (A) Spectra of ΔN6 (250 μM, red) and I_T_ (250 μM green), the latter obtained approximately 2 min after refolding Hβ_2_m from 8 M urea (pH 7.5, 25°C). (B) Panels showing the amide resonances of Glu69, Val93, and Asp96 in ΔN6 (red), native Hβ_2_m (black), and I_T_ (green), the latter obtained approximately 2 min, 5 min, 10 min, and 20 min after refolding commenced. (C) Comparison of the chemical shifts of ΔN6 and I_T_ for the 76 ^1^H or ^15^N resonances that were identified in the spectrum of both species. Note that the chemical shift differences of ΔN6 and I_T_ are more than one order of magnitude smaller than the chemical shift differences of Hβ_2_m and ΔN6 (compare Figure 1C with Figures 2B and 2D). Missing ^1^H-^15^N resonances in the spectrum of I_T_ (1–7, 29, 30, 33, 53, 55–61, 86, and 88) are either broadened due to intermediate exchange processes or degenerate with other residues. The rainbow ribbons and numbers above indicate β strands in Hβ_2_m calculated from the final set of 30 lowest-energy structures (PDB code 2XKS) using DSSPcont (Carter et al., 2003). All samples contained 0.8 M urea.

To confirm the structural similarity of ΔN6 with the slow-folding intermediate, I_T_, that is known to be highly amyloidogenic (Chiti et al., 2001; Jahn et al., 2006; Eichner and Radford, 2009), Hβ_2_m was denatured in 8 M urea and then refolded by 10-fold dilution into buffer at pH 7.5, 25°C. SOFAST ^1^H-^15^N heteronuclear multiple-quantum coherence (HMQC) spectra (Schanda and Brutscher, 2005) were then acquired approximately 2 min after refolding commenced, at which time I_T_ is populated to about 75% (Eichner and Radford, 2009) (Figure 1A). The spectrum reveals 76 cross-peaks corresponding to the I_T_ state, 68 of which overlay with resonances of ΔN6 (^1^H/^15^N within ± 0.04/0.2 ppm, respectively). Figure 1B shows one region of the SOFAST ^1^H-^15^N HMQC spectra obtained at different folding times. The data show that resonances arising from the kinetically formed I_T_ superpose with those of ΔN6 at equilibrium. The peaks corresponding to I_T_ then decrease in intensity with increased folding time, as resonances of the native state emerge. Resonances in the spectrum of ΔN6 were assigned using standard procedures (see below). Figure 1C shows the difference in chemical shift of the 76 cross-peaks identified for I_T_ by comparison with the assigned spectrum of ΔN6. The data reveal differences in chemical shift of <0.2 ppm (^1^H) or 0.8 ppm (^15^N), confirming the fidelity of ΔN6 as a structural mimic of I_T_.

### The Structure of ΔN6

**Figure 2:**
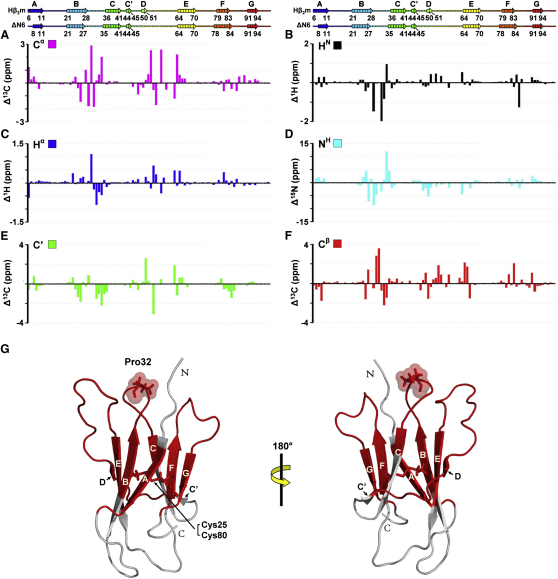
Chemical Shift Analysis of Native Hβ_2_m and ΔN6 (A–F) Differences in chemical shift of C^α^ (A), H^N^ (B), H^α^ (C), N^H^ (D), C′ (E), and C^β^ (F) between native Hβ_2_m and ΔN6 (pH 7.5, 25°C). Rainbow-colored ribbons and numbers above indicate the secondary structure contents of Hβ_2_m and ΔN6. (G) Lowest-energy structure of Hβ_2_m (PDB code 2XKS) showing the eight native β strands: A (6–11), B (21–28), C (36–41), C′ (44–45), D (50–51), E (64–70), F (79–83), and G (91–94). Residues colored in red differ significantly (Δ^1^H/^15^N/^13^C > 0.2/0.8/0.4 ppm) in chemical shift between Hβ_2_m and ΔN6. Pro32 (stick, spheres) is highlighted (see also Figure S1 and Table S1).

To allow detailed comparison of the structure and dynamics of ΔN6 and native Hβ_2_m, chemical shift assignment of the spectra of both proteins was carried out (Figure S1). ^1^H-^15^N assignments were obtained for 88 out of 99 residues of Hβ_2_m and 84 out of 93 residues of ΔN6. ^1^H-^13^C side-chain assignments were obtained for 95% of residues in Hβ_2_m and 90% of residues for ΔN6. The results indicate, as anticipated, that the X-Pro32 peptide bond adopts a *trans*-conformation in ΔN6 (Table S1). Moreover, comparison of the chemical shift differences between Hβ_2_m and ΔN6 (Figures 2A–2F) revealed that of the 93 residues in ΔN6, approximately 60 residues (i.e., more than half of the molecule) (Figure 2G) have chemical shifts that deviate substantially (Δ^1^H/^15^N/^13^C > 0.2/0.8/0.4 ppm for all resonances analyzed) from the values for native Hβ_2_m. While many of these residues lie in spatial proximity to Pro32 in native Hβ_2_m (Trinh et al., 2002), some are distant to this site, consistent with Pro32 being the epicenter of widespread conformational changes in ΔN6 (Figure 2G).

**Figure 3:**
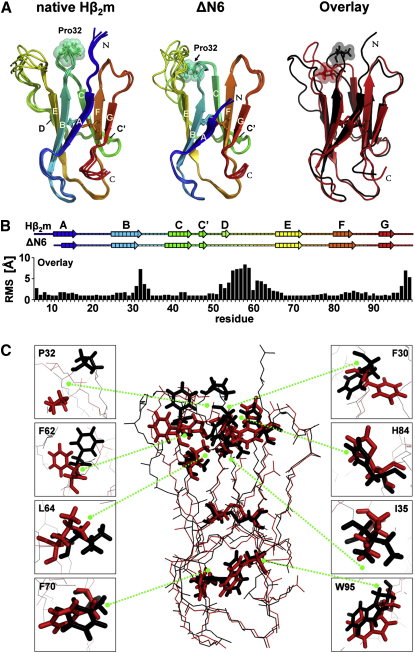
Solution Structures of Native Hβ_2_m and ΔN6 (A) Cartoon representation of five lowest-energy structures of native Hβ_2_m and ΔN6 with β strands highlighted in rainbow colors. The cartoon overlay shows the lowest-energy structures of native Hβ_2_m (black) and ΔN6 (red). Pro32 (sticks, spheres) and the disulfide bond (Cys25-Cys80, sticks) are highlighted. (B) Bar chart showing the Cα rms (Å) of the overlay shown in (A). The rainbow-colored arrows above indicate residues involved in β strand structure of Hβ_2_m and ΔN6. (C) Overlay of lowest-energy structures of native Hβ_2_m (black) and ΔN6 (red). Residues Pro32, Phe30, Phe62, His84, Leu64, Ile35, Phe70, and Trp95 and the disulfide bond (Cys25-Cys80, sticks) are highlighted. Structures were drawn using PyMOL (DeLano, 2002) (see also Figure S2 and Table S2).

To determine the structures of native Hβ_2_m and ΔN6, 2065 or 2565 nuclear Overhauser enhancements (nOe) (Tables 1 and S2), 128 or 118 dihedral angles, and 75 or 76 residual dipolar couplings (RDC) were obtained for each protein, respectively (Table 1). Structural ensembles were then calculated using the PASD algorithm (Kuszewski et al., 2004) to obtain 50 preliminary structures that were transferred into ARIA 2.0 (Rieping et al., 2007) for further refinement (see Supplemental Experimental Procedures). In the final ensemble of 30 structures (Figure 3A), the rmsd of backbone atoms (^1^H, ^15^N, ^13^Cα, ^13^C′) within ordered regions is 0.42 and 0.28 Å from the mean structures of native Hβ_2_m and ΔN6, respectively. The structural ensembles reveal that ΔN6 retains a native-like β sandwich fold containing two antiparallel β sheets tethered by a single disulfide bridge between Cys25 and Cys80 (Figure 3A). Minor differences are observed in the lengths of β strands, including the short D strand that adopts variable structures in ΔN6 (see Supplemental Experimental Procedures). Despite being highly amyloidogenic, therefore, ΔN6 contains a well-defined structure under the conditions employed that, in terms of the main chain, is not significantly perturbed compared with native Hβ_2_m (within ordered regions, the Cα rms between Hβ_2_m and ΔN6 is approximately 1.3 Å) (Figure 3B).

In contrast with the minor differences in the main chain of Hβ_2_m and ΔN6, dramatic differences are observed for side chains, both close to Pro32 and distal to this site, that result from a substantial repacking of the molecule's core (Figure 3C). Of the 21 residues that comprise the hydrophobic core of native Hβ_2_m, 17 undergo significant movement (>2 Å) between the two structures (Tables 1 and S2). Most strikingly, the aromatic side chain of Phe30 moves out of the hydrophobic core in ΔN6 toward the surface where the N terminus was originally placed (Figure 3C). This large movement (Hζ of Phe30 moves by ∼9.5 Å) is accompanied by further restructuring of side chains in the core (Figure 3C). In particular, the X-Pro32 *cis*-peptide bond is relaxed toward the more favored *trans*-peptidyl-prolyl isomer (Hγ moves by ∼9.6 Å). As a consequence, the backbone (^1^H, ^15^N, ^13^C′) interactions between Phe30 and Phe62 are disrupted so that the side chain of the latter rotates by >90° around Cγ and loses connection with the BC-loop (Cγ moves by ∼7.4 Å) (Figure 3C). The change in rotameric state of Phe30 also disrupts the main-chain hydrogen bonding between Ser28 and Lys6/Met6 in the A and B strands, leading to a loss in β strand structure at these residues and causing the side chain of His84 to rotate around its Cγ by ∼45° (Figure 3C). The cavity left by Phe30 is mostly filled by the hydrophobic side chains of Pro32, Leu64, and Ile35, the latter two side chains changing their rotamer angles so as to accomplish this. The conformational changes that result from isomerization of the X-Pro32 peptide bond do not stop at Cys25-Cys80 disulfide, but propagate deep into the other half of the molecule, leading to movements of the side chains of Asn21, Phe70, Phe78, and Trp95 (Figure 3C). These side-chain movements result in differences in surface charge and hydrophobicity (Figure S2A), consistent with previous suggestions that the N terminus of Hβ_2_m is important in maintaining the native hydrophobic folding balance (Esposito et al., 2000). The data thus show that docking of the N-terminal hexapeptide during the last steps in folding locks Hβ_2_m into a thermodynamically stable native structure that contains an unfavorable X-Pro32 *cis*-peptide bond. Consistent with this, resonances arising from the N-terminal seven residues were not identified in the HMQC spectrum of I_T_ (Figure 1A), suggesting that this region is displaced from its native conformation in the folding intermediate. Once the N-terminal hexapeptide is displaced, or removed as in ΔN6, the molecule relaxes toward an amyloidogenic conformation containing the X-Pro32 *trans*-isomer.

### The Dynamics of ΔN6 Reveal a Rarely Populated Nucleation- and Elongation-Competent Species

**Figure 4:**
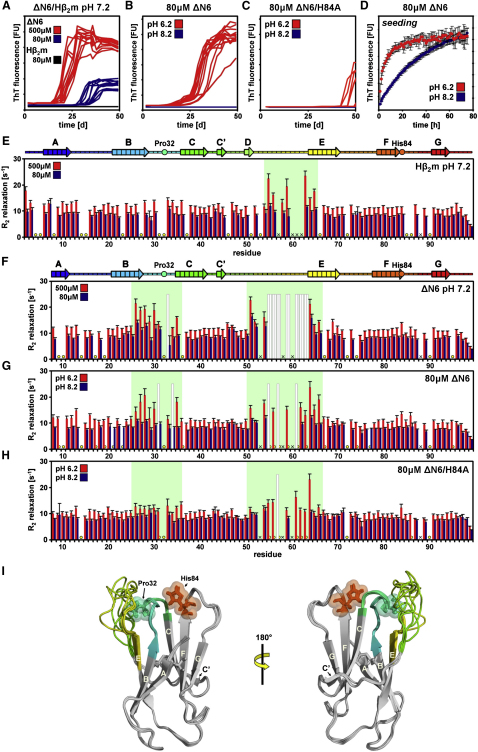
Amyloid Formation and Protein Dynamics of Native Hβ_2_m and ΔN6 (A) De novo fibril formation of 500 μM ΔN6 (red), 80 μM ΔN6 (blue), or 80 μM native Hβ_2_m (black) at pH 7.2, 37°C, 200 rpm. (B and C) De novo fibril assembly of 80 μM ΔN6 or ΔN6/H84A at pH 6.2 (red) or pH 8.2 (blue). (D) Seeded fibril assembly of 80 μM ΔN6 at pH 6.2 (red) or pH 8.2 (blue) using 10% (w/w) ΔN6 fibrillar seeds. The error bars are the standard deviation of six replicates. The presence of fibrillar material for all samples was confirmed by negative-stain electron microscopy (EM) (not shown). (E and F) ^15^N transverse relaxation measurements (R_2_ = 1/T_2_) of 500 μM (red) or 80 μM (blue) native Hβ_2_m or ΔN6 at pH 7.2, 25°C. Rainbow-colored ribbons above indicate the secondary structure content of native Hβ_2_m and ΔN6. (G and H) ^15^N transverse relaxation measurements of 80 μM ΔN6 or ΔN6/H84A at pH 6.2 or pH 8.2 (red and blue, respectively) at 25°C. Grey bars indicate a lower limit of the R_2_ for residues too weak to determine the value more precisely (>25 s^−1^). Circles highlight residues for which R_2_ could not be determined due to resonance overlap, line broadening, or the residue being a proline. Black crosses mark missing assignments. Green boxes highlight residues that show significant differences in local backbone dynamics in the different samples. The error (E–H) was estimated using duplicates. (I) Cartoon representation of five lowest-energy structures of ΔN6, highlighting Pro32 and His84 (sticks, spheres) and the regions that show enhanced local dynamics (residues 26–35 [dark green] and 51–66 [light green-yellow]) in the different samples. Note that both Hβ_2_m and ΔN6 show a uniform increase in R_2_ rates at higher protein concentrations, which are distinct from the pH-dependent enhanced R_2_ rates measured for residues in spatial proximity to Pro32 in ΔN6. The global increase in R_2_ rates observed for all residues in Hβ_2_m and ΔN6 at increased protein concentration most likely indicates transient oligomerization not associated with amyloid formation (see also Figure S3).

ΔN6 has been shown to be highly aggregation prone compared with Hβ_2_m, suggesting that this variant is uniquely able to sample one or more amyloidogenic conformers at physiological pH (Esposito et al., 2000; Eichner and Radford, 2009). Accordingly, amyloid fibrils are obtained after incubation of ΔN6 at 37°C, pH 7.2 in a protein-concentration-dependent manner (Figure 4A). While ΔN6 converts quantitatively into insoluble aggregates, as indicated by a lack of residual monomer in the supernatant after fibrillation is complete, as judged by SDS-PAGE (see Experimental Procedures) (data not shown), native Hβ_2_m does not show an increase in ThT fluorescence and remains soluble (judged by SDS-PAGE, data not shown) even after 50 days incubation (Figure 4A). Further experiments revealed that the amyloidogenicity of ΔN6 is highly pH dependent: no fibrils result when the protein (80 μM) is incubated at pH 8.2 for 50 days, while the protein converts into amyloid fibrils at pH 6.2 more rapidly (t_lag_ = 15 ± 4 days) than at pH 7.2 (t_lag_ = 35 ± 4 days) at this protein concentration (compare Figures 4A and 4B). Replacing His84 (a residue in close proximity to Pro32) (Figure 3C) with alanine in ΔN6 substantially reduces the ability of this protein to assemble de novo into amyloid fibrils (Figure 4C). The rate of elongation of fibrillar seeds of ΔN6 by ΔN6 monomers is also enhanced at pH 6.2 compared with pH 8.2 (Figure 4D). The data indicate, therefore, that protonation of His84 and possibly other side chains with pK_a_ ∼7 amplifies the amyloidogenicity of ΔN6, presumably by causing conformational changes that increase the population of species with enhanced amyloid potential within the ensemble of structures available.

To explore in more detail the enhanced ability of ΔN6 to nucleate amyloid formation compared with Hβ_2_m, the dynamic properties of the two proteins were compared. Whereas ^15^N R_1_ and {^1^H}^15^N nOe relaxation measurements show limited motions on a picosecond-to-nanosecond timescale for both proteins (Figure S2B), significantly higher ^15^N R_2_ relaxation rates were observed for residues 25–34 (BC-loop) in ΔN6 compared with Hβ_2_m (Figures 4E and 4F). Additionally, several residues in the DE-loop (54–57, 59, 61–63) of ΔN6 relax too rapidly (R_2_ ≥ 25 s^−1^, indicated by gray bars in Figures 4F–4H) to determine their R_2_ rates reliably but were readily quantified for Hβ_2_m, suggestive of enhanced chemical exchange processes on a microsecond-to-millisecond timescale for these residues in the former protein (Figures 4E and 4F). The local dynamics of residues 25–34 (BC-loop) and 51–66 (DE-loop) of ΔN6 are dependent on pH, with complete suppression of their enhanced dynamics at pH 8.2 and significant enhancement relative to other residues at pH 6.2 (Figure 4G), consistent with the pH dependency of fibril formation. Finally, the pH-dependent increase in R_2_ dynamics of residues 25–34 in ΔN6/H84A is substantially reduced at pH 6.2 compared with ΔN6 (Figures 4G and 4H), consistent with the view that protonation of His84 plays a role in enhancing the dynamics of the BC-loop (Figure 4I) and the amyloidogenicity of ΔN6. Analysis of ^1^H-^15^N HSQC spectra of ΔN6 at increased protein concentration revealed a set of resonances (in the BC- and DE-loops) that shifted significantly dependent on the protein concentration, suggesting that fibril formation of ΔN6 occurs by transient oligomerization via a newly formed dimerization interface involving these residues (Figures S3A and S3B). Importantly, a clear correlation is observed between chemical shift alterations of ΔN6 with pH and protein concentration (data not shown). These data suggest that protonation events and protein conformational changes are coupled processes that together initiate the aggregation cascade.

### ΔN6 Converts Native Hβ_2_m into an Amyloid-Competent State

**Figure 5:**
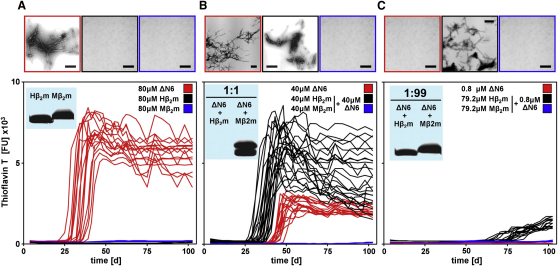
Enhancement and Inhibition of Hβ_2_m and ΔN6 Fibrillogenesis at pH 7.2 (37°C, 200 rpm) (A) ThT fluorescence of 80 μM ΔN6 (red), Hβ_2_m (black), or Mβ_2_m (blue). (B) ThT fluorescence of 40 μM ΔN6 incubated alone (red) or as mixtures of 40 μM Hβ_2_m and 40 μM ΔN6 (black) or 40 μM Mβ_2_m and 40 μM ΔN6 (blue). (C) ThT fluorescence of 0.8 μM ΔN6 (red) or mixtures of 79.2 μM Hβ_2_m and 0.8 μM ΔN6 (black) or 79.2 μM Mβ_2_m and 0.8 μM ΔN6 (blue). The upper panels show negative-stain EM images of the samples, using the same color coding. Scale bar = 100 nm. The insets (A–C) show SDS-PAGE analysis of the soluble fraction obtained after centrifugation (14,000 × *g*, 10 min) of the samples (see also Figure S5).

Given the inherent potential of ΔN6 to nucleate fibril formation in vitro, we speculated that bimolecular collision between ΔN6 and Hβ_2_m might enhance the amyloid potential of the latter protein by its conversion to an amyloidogenic state, akin to conformational conversion in prions. To test this hypothesis, Hβ_2_m and ΔN6 were incubated separately or as mixtures at a final total concentration of 80 μM in ratios of 1:1, 1:9, or 1:99 (ΔN6:Hβ_2_m) at pH 7.2, 37°C while shaking at 200 rpm (Figure 5 and data not shown). Whereas Hβ_2_m incubated alone does not form fibrils in the time frame of the experiment (100 days) (Figure 5A), consistent with previous results (Eichner and Radford, 2009), 50% (1:1), 10% (1:9), or even 1% (1:99) of ΔN6 is capable of catalyzing assembly of Hβ_2_m into amyloid-like fibrils with lag times of ∼30 ± 3, ∼40 ± 8, and ∼75 ± 8 days, respectively (Figures 5B and 5C and data not shown). Crucially, these lag times are shorter than those obtained when the same concentration of ΔN6 is incubated alone (∼40 ± 5 days, ∼50 ± 7 days, and >100 days [Figures 5B and 5C and data not shown]), indicating that productive interactions between ΔN6 and Hβ_2_m catalyze assembly of the latter into amyloid fibrils. As a control experiment, wild-type murine β_2_m (Mβ_2_m) was incubated alone or was mixed to a total protein concentration of 80 μM with different concentrations of ΔN6 (50% [1:1], 10% [1:9], and 1% [1:99]) (Figures 5A–5C). Mβ_2_m is 70% identical in sequence to Hβ_2_m but has been shown to be unable to assemble into amyloid fibrils at acidic pH (Ivanova et al., 2004). No fibrils were observed when Mβ_2_m was incubated alone at pH 7.2, 37°C for 100 days (Figure 5A). Remarkably, the presence of stoichiometric concentrations of Mβ_2_m abolishes the ability of ΔN6 to form amyloid fibrils (Figure 5B), and catalytic amounts (1%) of ΔN6 are not able to enhance amyloid formation from Mβ_2_m (Figure 5C). To confirm these findings, the amount of soluble material remaining in each sample was quantified using SDS-PAGE and fibril formation was monitored using EM (Figures 5A–5C). While all protein in 1:1 mixtures of ΔN6 and Mβ_2_m remained soluble (Figure 5B), 100%, ∼50%, and ∼10% conversion of Hβ_2_m into insoluble amyloid aggregates occurred when the protein was incubated with 50% (1:1), 10% (1:9), or 1% (1:99) ΔN6 (ΔN6:Hβ_2_m), respectively, consistent with the increased ThT fluorescence and the presence of fibrillar material in EM images of these samples (Figures 5B and 5C). The results indicate, therefore, that ΔN6 is able to convert Hβ_2_m into a conformer capable of forming amyloid fibrils even when added in catalytic amounts. Bimolecular collision of ΔN6 with the highly homologous Mβ_2_m protein, by contrast, abolishes the aggregation potential of the truncated protein.

### Atomistic Description of Conformational Conversion by Bimolecular Collision of ΔN6 and Hβ_2_m

**Figure 6:**
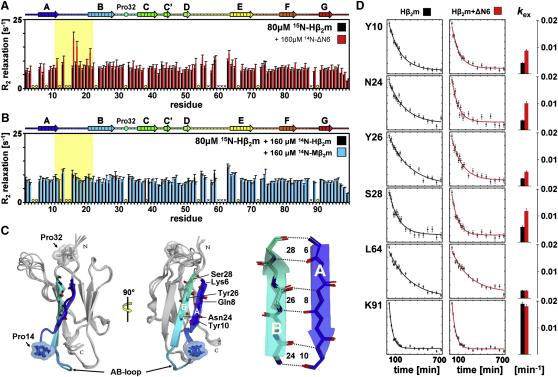
Atomistic Details of Specific Interactions between Native Hβ_2_m and ΔN6 (A and B) ^15^N transverse relaxation measurements (R_2_ = 1/T_2_) of 80 μM ^15^N-Hβ_2_m in the absence (black) or presence (red) of 160 μM ^14^N-ΔN6 at pH 6.2, 37°C, and 80 μM ^15^N-Hβ_2_m in the presence of 160 μM ^14^N-Hβ_2_m (black) or ^14^N-Mβ_2_m (blue) under the same conditions. Circles highlight residues for which data could not be obtained due to resonance overlap, line broadening, or the residue being proline. Black crosses mark missing assignments. Light yellow boxes emphasize residues 11–21 (AB-loop) that show increased backbone dynamics upon ΔN6 binding. The error was estimated using duplicates. (C) Five lowest-energy structures of native Hβ_2_m: Pro32 (light gray) and Pro14 (blue) are highlighted in sticks and spheres; the β strands A and B and the AB-loop are rainbow colored from blue to cyan. Residues that establish essential hydrogen bonds between β strands A and B (Lys6-Ser28, Gln8-Tyr26, Tyr10-Asn24) are highlighted in line representation alongside. (D) H-D exchange rates of 80 μM ^15^N-Hβ_2_m alone (black) or in the presence of 160 μM ^14^N-ΔN6 (red) at pH 6.2, 37°C. The black or red line is a single exponential fit of the data obtained. The error was estimated from the noise level of the experiment. The bars alongside depict the H-D exchange rate constant (k_ex_) of each residue, colored in the same manner (see also Figure S4).

To probe the molecular mechanism of conformational conversion of Hβ_2_m to an amyloidogenic state, backbone dynamics of ^15^N-Hβ_2_m in the absence or presence of ^14^N-ΔN6 (Figure 6A) were assessed using ^15^N NMR R_2_ measurements at pH 6.2, 37°C. These experiments revealed that adding ^14^N-ΔN6 to ^15^N-Hβ_2_m increases the ^15^N relaxation rates of residues 13–22 in the AB-loop of Hβ_2_m substantially (Figures 6A and 6C) in a pH-dependent manner (Figure S4A). Addition of equivalent concentrations of ^14^N-Hβ_2_m or ^14^N-Mβ_2_m has no effect on the R_2_ relaxation rates of ^15^N-Hβ_2_m (Figure 6B), indicating that the interaction of ΔN6 with Hβ_2_m is specifically able to alter the dynamics of the latter protein. Note that the enhanced R_2_ relaxation rates of residues in the AB-loop of Hβ_2_m in the presence of ΔN6 are not accompanied by ^1^H-^15^N chemical shift alterations (Figure S4B), implying that the concentration of the encounter complex between Hβ_2_m and ΔN6 is low (<5%) and its formation occurs on an intermediate NMR timescale (∼milliseconds).

The AB-loop (residues 13–22) (Figure 6C) has been shown to adopt different conformations in different crystal structures of Hβ_2_m, dependent on the contacts made in the crystal lattice (Ricagno et al., 2009). Additionally, it has been proposed that Pro14, which introduces rigidity into the AB-loop (Figure 6C), may play a role in triggering a conformational switch wherein the A strand is displaced toward a more open protein conformation (Rosano et al., 2004). As shown above, such a conformational transition would favor relaxation of the X-Pro32 *cis*-peptide bond to the *trans*-isomer by providing the conformational freedom required for the structural changes linked to this isomerization event to occur. To determine whether the increased dynamics of the AB-loop of Hβ_2_m induced by the presence of ΔN6 is linked to displacement of the A-strand, the hydrogen exchange (HX) rates of individual residues of ^15^N-Hβ_2_m, alone or mixed with a molar equivalent of ^14^N-ΔN6, were determined at pH 6.2, 37°C (Figure 6D). The results revealed a 2- to 3-fold increase in the HX rates of residues in the N-terminal region of Hβ_2_m in the presence of ^14^N-ΔN6 (exemplified by Tyr10, Asn24, Tyr26, and Ser28 in Figure 6D) compared to Hβ_2_m alone. By contrast, little effect (<1.5-fold) was observed for other residues in the protein (exemplified by Leu64 and Lys91 in Figure 6D). The data thus show that bimolecular collision of ΔN6 with Hβ_2_m increases the conformational dynamics of the N-terminal region of the protein, which permits the isomerization of the X-Pro32 bond and switches on the pathway toward the amyloid state.

## Discussion

### Role of the N Terminus in the Folding and Stabilization of Hβ_2_m

A key question in understanding amyloidosis is the nature of the early conformational changes that tip the equilibrium from correct folding toward the population of amyloidogenic species. Furthermore, how bimolecular collisions between a native protein and a misfolded or nonnative state enable conversion of an initially innocuous protein into an amyloidogenic conformation remains poorly understood at a molecular level. Here, we have used ΔN6 to investigate these phenomena. The solution structure of ΔN6 shows that this amyloidogenic protein retains a native-like structure, revealing that structural considerations alone cannot explain the enhanced amyloidogenic potential of this variant. Examination of the solution structures of native Hβ_2_m and ΔN6, nonetheless, reveals significant side-chain rearrangements comprising more than half of the protein's core that occur when the X-Pro32 peptide bond isomerizes to its *trans*-state, some of which involve residues shown hitherto to undergo structural changes in conformers of β_2_m trapped in protein crystals (Eakin et al., 2006; Calabrese et al., 2008). Despite the structural similarities of ΔN6 in solution and Cu^2+^-bound β_2_m observed crystallographically (Figures S2C–S2E), only ΔN6 is able to form amyloid fibrils efficiently in vitro (Eichner and Radford, 2009), suggesting that differences in structure and/or dynamics of these species are critical in endowing the potential to form amyloid.

**Figure 7:**
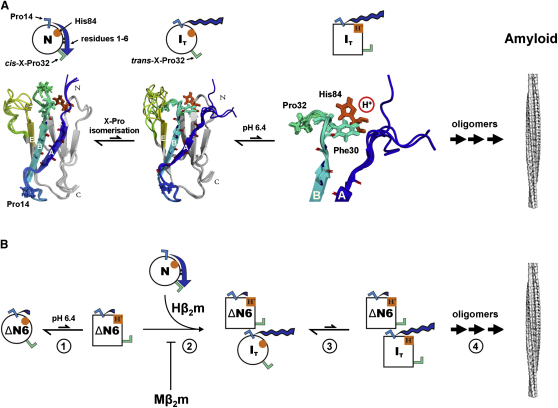
Turning On and Off β_2_m Amyloid Assembly (A) Summary showing the structures of Hβ_2_m and a model of I_T_. Above, keys for these conformational states. Native Hβ_2_m (leftmost), shown above as a circle with *cis*-X-Pro32 (green **Γ**), *trans*-X-Pro14 (blue **Γ**), His84 (red circle), and the N-terminal region (residues 1–6, in blue). Upon dissociation of the N-terminal region, the X-Pro32 bond is free to relax into the *trans*-conformation, causing further conformational changes that lead to the formation of the nonnative I_T_ conformer (shown as a circle above a model of its structure). Protonation of His84 (shown in red and as a red circle above), which lies adjacent to Pro32 under mildly acidic conditions, further enhances the amyloid potential of I_T_. Oligomerization of these aggregation-prone species then leads to the formation of β_2_m amyloid fibrils. Assuming that the fibrils formed at neutral pH are structurally similar to those formed at acidic pH, as suggested by FTIR (Jahn et al., 2008) and solid-state NMR (Debelouchina et al., 2010), large conformational changes are required in order to transform the antiparallel β sheet arrangement of ΔN6 into the parallel in-register arrangement of β strands characteristic of β_2_m amyloid fibrils, as reported recently (Ladner et al., 2010). (B) Proposed mechanism of conformational conversion of Hβ_2_m into an amyloid-competent state by bimolecular collision with ΔN6. ΔN6 (shown schematically as a circle, using similar color schemes as in A) lacks the N-terminal six residues that clamp Hβ_2_m into its native structure. Protonation of His84 (shown as a red circle [unprotonated] or square [protonated]) occurs at mildly acidic pH. Possibly accompanied by alterations in the conformation/protonation status of other residues, the amyloidogenicity of ΔN6 is enhanced (Step 1). Bimolecular collision of one or more rarely populated conformers of ΔN6 with native Hβ_2_m leads to dissociation of the N-terminal region of the latter protein, allowing *cis*-to-*trans* isomerization of X-Pro32 to occur (Step 2). Subsequent protonation of His84 in the wild-type protein is then favored, completing conformational conversion of I_T_ (Step 3). Further protein-protein interactions between these species then allow nucleation and elongation of amyloid fibrils (Step 4).

While ΔN6 retains a native topology and is only marginally destabilized compared with Hβ_2_m (ΔΔG°_UN_ = 3.8 kJ mol^−1^) (Eichner and Radford, 2009), removal of the N-terminal six residues disrupts the kinetic stability of the protein, such that it is no longer strongly protected from HX (Figure S3C) and interconverts rapidly with other conformers on a microsecond-to-millisecond timescale, as revealed by its enhanced R_2_ values compared with Hβ_2_m (Figures 4E and 4F). Furthermore, the absence of well-resolved resonances for residues 1–7 in I_T_ (Figure 1A) suggests that these residues are not natively attached to the protein in this folding intermediate. The N-terminal residues of β_2_m (Figure 7A, shown in blue) thus act as a postassembly clamp, side chains in this region stabilizing residues in the BC-loop and its main chain forming hydrogen bonds to the adjacent native β strand B. These interactions lock the X-Pro32 peptide bond in the *cis*-isomer, preventing its isomerization to the relaxed *trans*-form and the consequent release of the side chain of Phe30 from the hydrophobic core that, together, initiate a cascade of events that opens up the pathway toward aggregation. Proline-mediated loop dynamics associated with protein assembly have been observed in a number of protein systems in vitro and in vivo, suggesting that proline-mediated triggering of amyloidosis is not unique to β_2_m. For example, a misfolded conformer of human immunoglobulin light chain (O'Nuallain et al., 2007), an amyloid intermediate of stefin B (Jenko Kokalj et al., 2007), and the cell-cycle protein p13suc1 (Rousseau et al., 2001) have all been shown to possess aggregation mechanisms dependent on isomerization of X-Pro bonds.

### ΔN6: An In Vivo Culprit of DRA

Despite the fact that β_2_m is among the most extensively studied proteins involved in human amyloid disease (Platt and Radford, 2009), the initiating factors in DRA remain unclear. Many scenarios have been suggested, including partial unfolding of β_2_m on the collagen surface, mild acidification in arthritic joints, stabilization of rare fibrils that may form from Hβ_2_m by glycosamingoglycans (Esposito et al., 2000; Yamamoto et al., 2004b; Eakin and Miranker, 2005; Mimmi et al., 2006; Myers et al., 2006; Relini et al., 2006), as well as mechanisms involving the addition of metal ions, lipids, or other factors that enhance the initial unfolding events required for assembly to occur (Yamamoto et al., 2004a; Eakin and Miranker, 2005; Ohhashi et al., 2005; Sasahara et al., 2008). Here, we have shown that ΔN6 is a highly amyloidogenic species that is able to nucleate fibrillogenesis efficiently in vitro at neutral pH. This observation rationalizes the lack of circulating ΔN6 in the serum of patients with renal dysfunction and, given the natural affinity of ΔN6 for collagen (which is enhanced relative to Hβ_2_m [Giorgetti et al., 2005]), explains why assembly of fibrils occurs most readily in collagen-rich joints. Additionally, we show that the mild acidification, such as may occur in the synovial fluid of patients undergoing long-term hemodialysis (Relini et al., 2006), has a dramatic effect in enhancing fibril formation of ΔN6 and its ability to convert Hβ_2_m into an amyloid-competent state by protonation of His84 and/or other residues in the protein. We propose that rather than being an innocuous postassembly event (Monti et al., 2005), proteolytic cleavage of the N-terminal region of β_2_m could be a key initiating event in DRA. Such cleavage enables the formation of species that are not only able to assemble de novo into amyloid fibrils, which thereafter can enhance fibrillogenesis of Hβ_2_m by cross-seeding (Figure S5), but when present in only catalytic amounts are also able to convert the wild-type protein into an amyloidogenic state (Figure 5C).

### Conformational Conversion in Atomistic Detail

A fascinating feature of amyloid fibrils is their ability to consume soluble monomer for self-replication via elongation with their own, or closely related, protein monomers. This suggests that amyloid fibrils or misfolded protein aggregates in general may have an inherent potential to convert innocuous protein conformers into amyloidogenic species, a feature famously associated with prions (Sindi and Serio, 2009). By exploring the dynamics and aggregation behavior of ΔN6, this study reveals that conformational excursions of ΔN6, which occur more frequently upon mild acidification, not only rationalize the inherent amyloidogenicity of this protein but also explain how this species is able to convert Hβ_2_m into an amyloidogenic state. By contrast, transient bimolecular collision between ΔN6 and Mβ_2_m abolishes the ability of ΔN6 to convert into amyloid fibrils.

NMR analysis of bimolecular collisions between ΔN6 and Hβ_2_m (Figures 6A–6D) exposes atomistic details of one possible route of conformational conversion during amyloid formation (Figure 7B). First, protonation of His84 and/or other amino acid side chains of ΔN6 under mild acidification enhances the aggregation potential of this already amyloidogenic protein (Step 1, Figure 7B). Next, specific bimolecular collision between ΔN6 and Hβ_2_m alters the dynamic properties of the AB-loop in Hβ_2_m that contains Pro14, which leads to partial fraying or displacement of the A-strand from the native β sandwich, with concomitant loss of HX protection in this region (Step 2). By this mechanism the X-Pro32 peptide bond becomes free to relax to the *trans*-isomer, triggering the cascade of events involved in the conversion of the constrained native protein to a dynamic amyloidogenic state (Step 3). Further assembly of monomers then leads to the formation of amyloid fibrils via one or more oligomeric species (Step 4). Correct docking of the A strand is thus crucial in trapping the native protein into a unique conformation and maintaining the concentration of amyloidogenic precursor states below that required for amyloid formation. Exploiting the power of NMR, we reveal here in atomistic detail how a nonnative protein conformer is able to convert an originally innocuous native protein species into an amyloidogenic state, opening the door to protein self-assembly and the onset of amyloidosis.

## Experimental Procedures

### Protein Preparation

Proteins were prepared as previously described (Platt et al., 2005).

### Assembly of Amyloid Fibrils

Samples were prepared in 96-well plates (Corning Incorporated, Costar) containing 0.8–500 μM protein, 81–89.5 mM NaCl, 10 μM ThT, and 0.02% (w/v) sodium azide in 10 mM sodium phosphate buffer (pH 6.2–8.2). Seeded reactions contained additionally 10% (w/w) ΔN6 seeds (see Supplemental Experimental Procedures). De novo fibril growth was performed by incubating the 96-well plate at 37°C, 200 rpm, while seeded reactions were carried out quiescently at 37°C. ThT fluorescence (excitation 440 nm, emission 480 nm) was measured using a plate reader (FLUOstar OPTIMA) at 37°C. The soluble fraction obtained after centrifugation (14,000 × *g*, 10 min) was analyzed by SDS-PAGE.

### NMR Spectroscopy and Structure Determination

Samples of ^15^N- or ^13^C-^15^N-labeled protein (0.3–1.0 mM) in 25 mM sodium phosphate buffer pH 7.5, 0.02% (w/v) sodium azide, 90% (v/v) H_2_O/10% (v/v) D_2_O were used for NMR experiments.

Spectra were recorded at 25°C on a Varian Inova 500 MHz or 600 MHz instrument with a room temperature probe or a Varian Inova 750 MHz spectrometer equipped with a cryogenic probe. Sequential assignment, structural restraint, structure calculation, and other NMR procedures are detailed in the Supplemental Experimental Procedures online.

### ^15^N NMR Relaxation, Saturation Transfer, and HX Experiments

Backbone ^15^N transverse relaxation (R_2_ = 1/T_2_), ^15^N longitudinal relaxation (R_1_ = 1/T_1_), {^1^H}^15^N nOe relaxation measurements, and measurements of HX were carried out as described (Farrow et al., 1994; Gorski et al., 2004). Further details are given in the Supplemental Experimental Procedures online.

## Figures and Tables

**Table 1:** NMR and Refinement Statistics of Native Hβ_2_m and ΔN6 (pH 7.5, 25°C)

*NMR Distance and Dihedral Constraints*	Native Hβ_2_m	ΔN6
**Distance Constraints**		

Total nOe	2065	2565
Intraresidue	782	732
Interresidue	1283	1833
Sequential (|i-j| = 1)	412	526
Medium-range (|i-j| ≤ 4)	173	286
Long-range (|i-j| ≥ 5)	698	1021
Intermolecular	0	0
Total RDCs	75	76
^1^D_HN_	75	76

**Total Dihedral Angle Restraints**	**128**	**118**

Φ	64	59
Ψ	64	59

***Structure Statistics***	**Native Hβ_2_m**	**ΔN6**

**Violations (Mean and SD)**		

Distance constraints (Å)	0.155 ± 0.007	0.056 ± 0.005
Dihedral angle constraints (°)	1.29 ± 0.24	1.07 ± 0.33
Max. distance constraint violation (Å)	0.045	0.050
Max. dihedral angle violation (°)	6.9	<6.0
RDC Q	0.33 ± 0.22	0.43 ± 0.24
Average RDC violation (°)	0.64 ± 0.06	0.78 ± 0.06

**Deviations from Idealized Geometry**		

Bond length (Å)	0.0056 ± 0.0002	0.0060 ± 0.0001
Bond angle (°)	0.72 ± 0.02	0.82 ± 0.02
Impropers (°)	1.87 ± 0.11	2.05 ± 0.15

**Average Pairwise Rmsd**∗**(Å)**		

Heavy	1.218	0.984
Backbone	0.415	0.277

∗ Pairwise rmsds were calculated over ordered regions from an ensemble of 30 structures superimposing the restrained residues in native Hβ_2_m (3–28, 30–31, 34–45, 48–53, 55–57, 63–86, 89–98) and ΔN6 (7–28, 31–52, 64–86, 89–98).

## Accession Numbers

Coordinates and structural restraints of Hβ_2_m and ΔN6 have been deposited in the PDB and chemical shifts, peak lists, and rdc measurements in BMRB with accession numbers 2XKS and 17165 or 2XKU and 17166, respectively.

## Supplemental Information

Document S1. Supplemental Experimental Procedures, Supplemental References, Five Figures, and Two Tables

## References

[bib1] Ahn H.C., Le Y.T., Nagchowdhuri P.S., Derose E.F., Putnam-Evans C., London R.E., Markley J.L., Lim K.H. (2006). NMR characterizations of an amyloidogenic conformational ensemble of the PI3K SH3 domain. Protein Sci..

[bib2] Angot E., Brundin P. (2009). Dissecting the potential molecular mechanisms underlying alpha-synuclein cell-to-cell transfer in Parkinson's disease. Parkinsonism Relat. Disord..

[bib3] Becker J.W., Reeke G.N. (1985). Three-dimensional structure of beta 2-microglobulin. Proc. Natl. Acad. Sci. USA.

[bib4] Booth D.R., Sunde M., Bellotti V., Robinson C.V., Hutchinson W.L., Fraser P.E., Hawkins P.N., Dobson C.M., Radford S.E., Blake C.C., Pepys M.B. (1997). Instability, unfolding and aggregation of human lysozyme variants underlying amyloid fibrillogenesis. Nature.

[bib5] Brundin P., Melki R., Kopito R. (2010). Prion-like transmission of protein aggregates in neurodegenerative diseases. Nat. Rev. Mol. Cell Biol..

[bib6] Calabrese M.F., Miranker A.D. (2009). Metal binding sheds light on mechanisms of amyloid assembly. Prion.

[bib7] Calabrese M.F., Eakin C.M., Wang J.M., Miranker A.D. (2008). A regulatable switch mediates self-association in an immunoglobulin fold. Nat. Struct. Mol. Biol..

[bib8] Calamai M., Chiti F., Dobson C.M. (2005). Amyloid fibril formation can proceed from different conformations of a partially unfolded protein. Biophys. J..

[bib9] Carter P., Andersen C.A., Rost B. (2003). DSSPcont: Continuous secondary structure assignments for proteins. Nucleic Acids Res..

[bib10] Chiti F., De Lorenzi E., Grossi S., Mangione P., Giorgetti S., Caccialanza G., Dobson C.M., Merlini G., Ramponi G., Bellotti V. (2001). A partially structured species of beta 2-microglobulin is significantly populated under physiological conditions and involved in fibrillogenesis. J. Biol. Chem..

[bib11] Corazza A., Rennella E., Schanda P., Mimmi M.C., Cutuil T., Raimondi S., Giorgetti S., Fogolari F., Viglino P., Frydman L. (2010). Native-unlike long-lived intermediates along the folding pathway of the amyloidogenic protein beta2-microglobulin revealed by real-time two-dimensional NMR. J. Biol. Chem..

[bib12] Debelouchina G.T., Platt G.W., Bayro M.J., Radford S.E., Griffin R.G. (2010). Magic angle spinning NMR analysis of beta2-microglobulin amyloid fibrils in two distinct morphologies. J. Am. Chem. Soc..

[bib13] DeLano W.L. (2002). The PyMOL Molecular Graphics System. http://www.pymol.org/.

[bib14] Eakin C.M., Miranker A.D. (2005). From chance to frequent encounters: origins of beta2-microglobulin fibrillogenesis. Biochim. Biophys. Acta.

[bib15] Eakin C.M., Berman A.J., Miranker A.D. (2006). A native to amyloidogenic transition regulated by a backbone trigger. Nat. Struct. Mol. Biol..

[bib16] Eichner T., Radford S.E. (2009). A generic mechanism of beta2-microglobulin amyloid assembly at neutral pH involving a specific proline switch. J. Mol. Biol..

[bib17] Esposito G., Michelutti R., Verdone G., Viglino P., Hernandez H., Robinson C.V., Amoresano A., Dal Piaz F., Monti M., Pucci P. (2000). Removal of the N-terminal hexapeptide from human beta2-microglobulin facilitates protein aggregation and fibril formation. Protein Sci..

[bib18] Farrow N.A., Muhandiram R., Singer A.U., Pascal S.M., Kay C.M., Gish G., Shoelson S.E., Pawson T., Forman-Kay J.D., Kay L.E. (1994). Backbone dynamics of a free and phosphopeptide-complexed Src homology 2 domain studied by 15N NMR relaxation. Biochemistry.

[bib19] Gejyo F., Yamada T., Odani S., Nakagawa Y., Arakawa M., Kunitomo T., Kataoka H., Suzuki M., Hirasawa Y., Shirahama T. (1985). A new form of amyloid protein associated with chronic hemodialysis was identified as beta2-microglobulin. Biochem. Biophys. Res. Commun..

[bib20] Gidalevitz T., Ben-Zvi A., Ho K.H., Brignull H.R., Morimoto R.I. (2006). Progressive disruption of cellular protein folding in models of polyglutamine diseases. Science.

[bib21] Giorgetti S., Rossi A., Mangione P., Raimondi S., Marini S., Stoppini M., Corazza A., Viglino P., Esposito G., Cetta G. (2005). Beta2-microglobulin isoforms display an heterogeneous affinity for type I collagen. Protein Sci..

[bib22] Gorski S.A., Le Duff C.S., Capaldi A.P., Kalverda A.P., Beddard G.S., Moore G.R., Radford S.E. (2004). Equilibrium hydrogen exchange reveals extensive hydrogen bonded secondary structure in the on-pathway intermediate of Im7. J. Mol. Biol..

[bib23] Ivanova M.I., Sawaya M.R., Gingery M., Attinger A., Eisenberg D. (2004). An amyloid-forming segment of beta2-microglobulin suggests a molecular model for the fibril. Proc. Natl. Acad. Sci. USA.

[bib24] Jahn T.R., Radford S.E. (2008). Folding versus aggregation: polypeptide conformations on competing pathways. Arch. Biochem. Biophys..

[bib25] Jahn T.R., Parker M.J., Homans S.W., Radford S.E. (2006). Amyloid formation under physiological conditions proceeds via a native-like folding intermediate. Nat. Struct. Mol. Biol..

[bib26] Jahn T.R., Tennent G.A., Radford S.E. (2008). A common beta-sheet architecture underlies in vitro and in vivo beta2-microglobulin amyloid fibrils. J. Biol. Chem..

[bib27] Jenko Kokalj S., Guncar G., Stern I., Morgan G., Rabzelj S., Kenig M., Staniforth R.A., Waltho J.P., Zerovnik E., Turk D. (2007). Essential role of proline isomerization in stefin B tetramer formation. J. Mol. Biol..

[bib28] Kameda A., Morita E.H., Sakurai K., Naiki H., Goto Y. (2009). NMR-based characterization of a refolding intermediate of beta2-microglobulin labeled using a wheat germ cell-free system. Protein Sci..

[bib29] Kane M.D., Lipinski W.J., Callahan M.J., Bian F., Durham R.A., Schwarz R.D., Roher A.E., Walker L.C. (2000). Evidence for seeding of beta -amyloid by intracerebral infusion of Alzheimer brain extracts in beta -amyloid precursor protein-transgenic mice. J. Neurosci..

[bib30] Kuszewski J., Schwieters C.D., Garrett D.S., Byrd R.A., Tjandra N., Clore G.M. (2004). Completely automated, highly error-tolerant macromolecular structure determination from multidimensional nuclear overhauser enhancement spectra and chemical shift assignments. J. Am. Chem. Soc..

[bib31] Ladner C.L., Chen M., Smith D.P., Platt G.W., Radford S.E., Langen R. (2010). Stacked sets of parallel, in-register beta-strands of beta2-microglobulin in amyloid fibrils revealed by site-directed spin labeling and chemical labeling. J. Biol. Chem..

[bib32] Liu K., Cho H.S., Lashuel H.A., Kelly J.W., Wemmer D.E. (2000). A glimpse of a possible amyloidogenic intermediate of transthyretin. Nat. Struct. Biol..

[bib33] Lundmark K., Westermark G.T., Nystrom S., Murphy C.L., Solomon A., Westermark P. (2002). Transmissibility of systemic amyloidosis by a prion-like mechanism. Proc. Natl. Acad. Sci. USA.

[bib34] Mimmi M.C., Jorgensen T.J., Pettirossi F., Corazza A., Viglino P., Esposito G., De Lorenzi E., Giorgetti S., Pries M., Corlin D.B. (2006). Variants of beta-microglobulin cleaved at lysine-58 retain the main conformational features of the native protein but are more conformationally heterogeneous and unstable at physiological temperature. FEBS J..

[bib35] Monti M., Amoresano A., Giorgetti S., Bellotti V., Pucci P. (2005). Limited proteolysis in the investigation of beta2-microglobulin amyloidogenic and fibrillar states. Biochim. Biophys. Acta.

[bib36] Myers S.L., Jones S., Jahn T.R., Morten I.J., Tennent G.A., Hewitt E.W., Radford S.E. (2006). A systematic study of the effect of physiological factors on beta2-microglobulin amyloid formation at neutral pH. Biochemistry.

[bib37] O'Nuallain B., Allen A., Kennel S.J., Weiss D.T., Solomon A., Wall J.S. (2007). Localization of a conformational epitope common to non-native and fibrillar immunoglobulin light chains. Biochemistry.

[bib38] Ohhashi Y., Kihara M., Naiki H., Goto Y. (2005). Ultrasonication-induced amyloid fibril formation of beta2-microglobulin. J. Biol. Chem..

[bib39] Platt G.W., Radford S.E. (2009). Glimpses of the molecular mechanisms of beta2-microglobulin fibril formation in vitro: aggregation on a complex energy landscape. FEBS Lett..

[bib40] Platt G.W., McParland V.J., Kalverda A.P., Homans S.W., Radford S.E. (2005). Dynamics in the unfolded state of beta2-microglobulin studied by NMR. J. Mol. Biol..

[bib41] Qin Z., Hu D., Zhu M., Fink A.L. (2007). Structural characterization of the partially folded intermediates of an immunoglobulin light chain leading to amyloid fibrillation and amorphous aggregation. Biochemistry.

[bib42] Relini A., Canale C., De Stefano S., Rolandi R., Giorgetti S., Stoppini M., Rossi A., Fogolari F., Corazza A., Esposito G. (2006). Collagen plays an active role in the aggregation of beta2-microglobulin under physiopathological conditions of dialysis-related amyloidosis. J. Biol. Chem..

[bib43] Ricagno S., Raimondi S., Giorgetti S., Bellotti V., Bolognesi M. (2009). Human beta-2 microglobulin W60V mutant structure: Implications for stability and amyloid aggregation. Biochem. Biophys. Res. Commun..

[bib44] Richardson J.S., Richardson D.C. (2002). Natural beta-sheet proteins use negative design to avoid edge-to-edge aggregation. Proc. Natl. Acad. Sci. USA.

[bib45] Rieping W., Habeck M., Bardiaux B., Bernard A., Malliavin T.E., Nilges M. (2007). ARIA2: automated NOE assignment and data integration in NMR structure calculation. Bioinformatics.

[bib46] Rocken C., Shakespeare A. (2002). Pathology, diagnosis and pathogenesis of AA amyloidosis. Virchows Arch..

[bib47] Rosano C., Zuccotti S., Mangione P., Giorgetti S., Bellotti V., Pettirossi F., Corazza A., Viglino P., Esposito G., Bolognesi M. (2004). beta2-microglobulin H31Y variant 3D structure highlights the protein natural propensity towards intermolecular aggregation. J. Mol. Biol..

[bib48] Rousseau F., Schymkowitz J.W., Wilkinson H.R., Itzhaki L.S. (2001). Three-dimensional domain swapping in p13suc1 occurs in the unfolded state and is controlled by conserved proline residues. Proc. Natl. Acad. Sci. USA.

[bib49] Sakata M., Chatani E., Kameda A., Sakurai K., Naiki H., Goto Y. (2008). Kinetic coupling of folding and prolyl isomerization of beta2-microglobulin studied by mutational analysis. J. Mol. Biol..

[bib50] Sasahara K., Yagi H., Sakai M., Naiki H., Goto Y. (2008). Amyloid nucleation triggered by agitation of beta2-microglobulin under acidic and neutral pH conditions. Biochemistry.

[bib51] Schanda P., Brutscher B. (2005). Very fast two-dimensional NMR spectroscopy for real-time investigation of dynamic events in proteins on the time scale of seconds. J. Am. Chem. Soc..

[bib52] Sindi S.S., Serio T.R. (2009). Prion dynamics and the quest for the genetic determinant in protein-only inheritance. Curr. Opin. Microbiol..

[bib53] Trinh C.H., Smith D.P., Kalverda A.P., Phillips S.E., Radford S.E. (2002). Crystal structure of monomeric human beta-2-microglobulin reveals clues to its amyloidogenic properties. Proc. Natl. Acad. Sci. USA.

[bib54] Welch W.J. (2004). Role of quality control pathways in human diseases involving protein misfolding. Semin. Cell Dev. Biol..

[bib55] Westermark P., Benson M.D., Buxbaum J.N., Cohen A.S., Frangione B., Ikeda S., Masters C.L., Merlini G., Saraiva M.J., Sipe J.D. (2007). A primer of amyloid nomenclature. Amyloid.

[bib56] Xing Y., Nakamura A., Korenaga T., Guo Z., Yao J., Fu X., Matsushita T., Kogishi K., Hosokawa M., Kametani F. (2002). Induction of protein conformational change in mouse senile amyloidosis. J. Biol. Chem..

[bib57] Yamamoto S., Hasegawa K., Yamaguchi I., Tsutsumi S., Kardos J., Goto Y., Gejyo F., Naiki H. (2004). Low concentrations of sodium dodecyl sulfate induce the extension of beta 2-microglobulin-related amyloid fibrils at a neutral pH. Biochemistry.

[bib58] Yamamoto S., Yamaguchi I., Hasegawa K., Tsutsumi S., Goto Y., Gejyo F., Naiki H. (2004). Glycosaminoglycans enhance the trifluoroethanol-induced extension of beta 2-microglobulin-related amyloid fibrils at a neutral pH. J. Am. Soc. Nephrol..

