# Supplementary material for: Conformational Conversion during Amyloid Formation at Atomic Resolution
Source: Mol Cell. 2011 Jan 21;41(2-4):161–72. doi: 10.1016/j.molcel.2010.11.028 (PMC3029554; doi:10.1016/j.molcel.2010.11.028)
Supplement: Document S1. Supplemental Experimental Procedures, Supplemental References, Five Figures, and Two Tables [file mmc1.pdf]

Supplemental Information
Molecular Cell, *Volume 41*

Conformational Conversion during Amyloid Formation at Atomic Resolution

Timo Eichner, Arnout P. Kalverda, Gary S. Thompson, Steve W. Homans, Sheena E. Radford

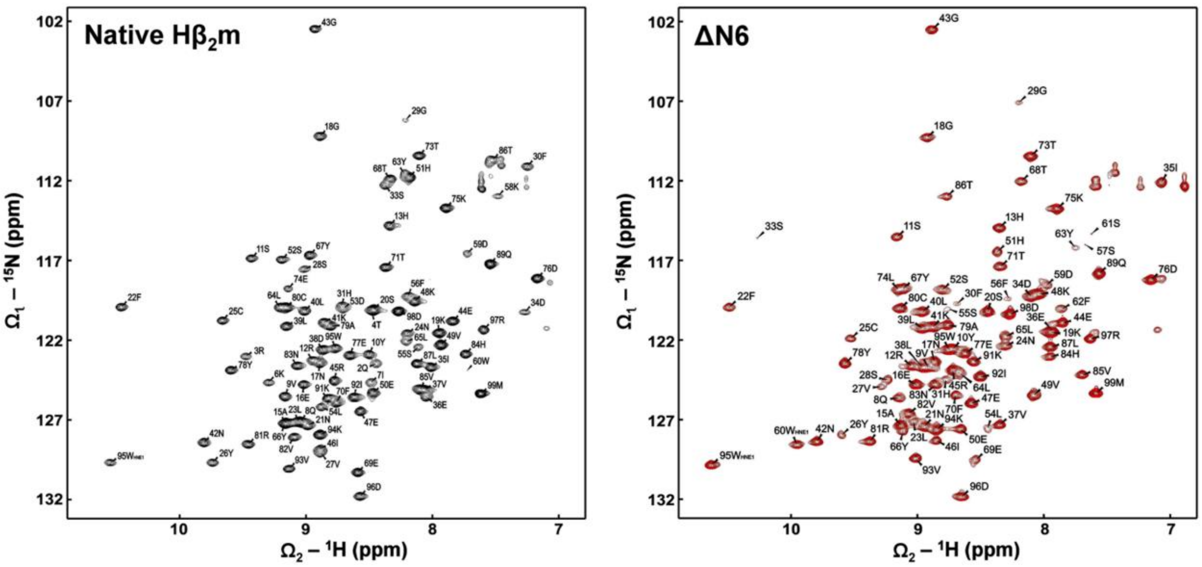

Figure S1, related to Figure 2. ^{1}H-^{15}N HSQC Spectra of Native Hβ$_2$m and ΔN6 (each 80 μM) Obtained at pH 7.5, 25 °C

Missing assignments for native Hβ$_2$m (residues 1, 57, 60-62, 88) and for ΔN6 (residues 7, 53, 58, 60, 88) suggest these resonances are either weak in intensity due to line broadening or have chemical shifts that are degenerate with other resonances.

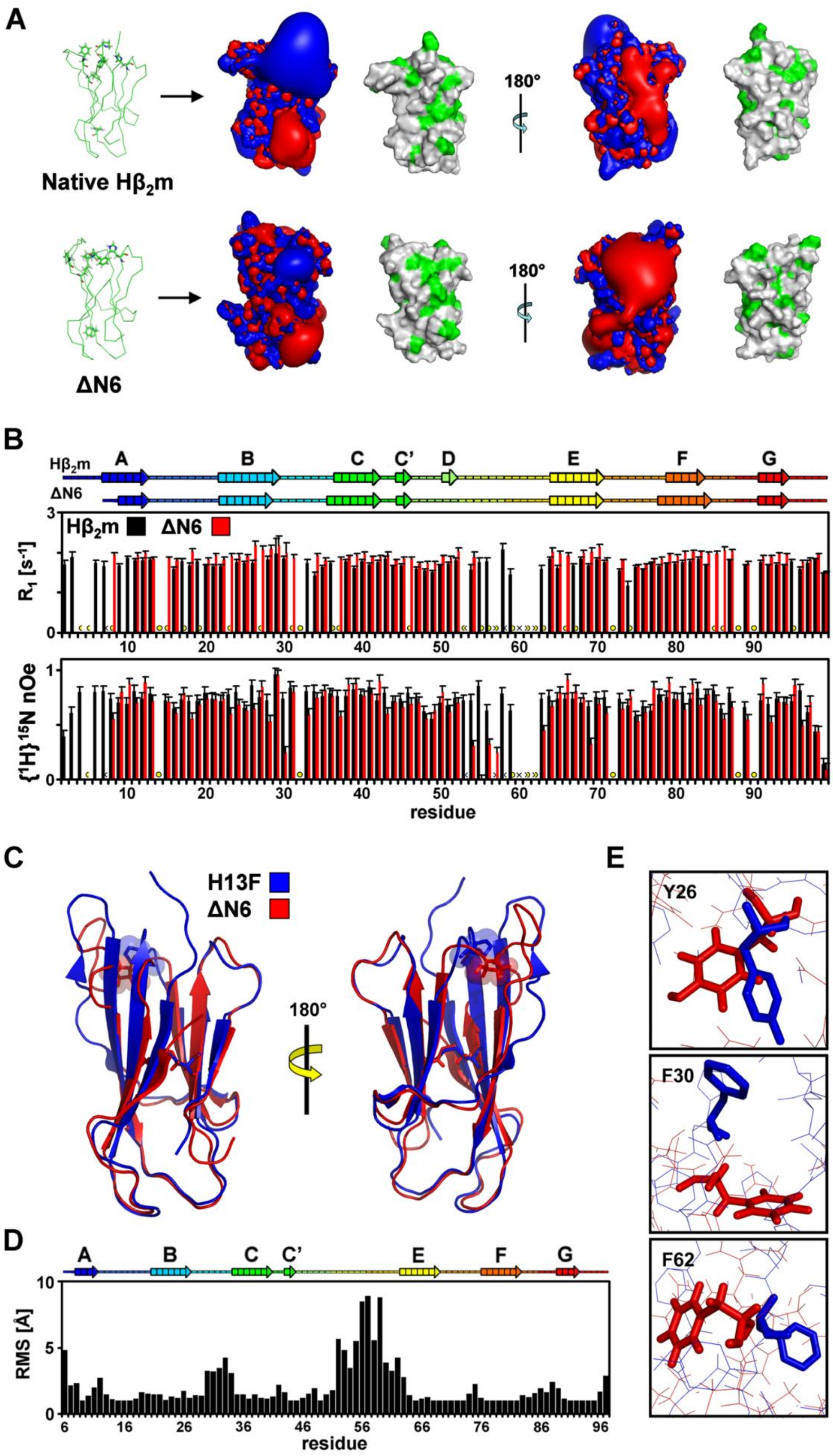
A
Native Hβ2m
180°
ΔN6
180°
B
Hβ2m
ΔN6
A B C C' D E F G
Hβ2m ΔN6
R1 [s-1]
{1H}15N nOe
residue
C
H13F
ΔN6
180°
D
A B C C' E F G
RMS [Å]
residue
E
Y26
F30
F62

Figure S2, related to Figure 3. Comparison of the Structures and Dynamics of Native Hβ_2m, ΔN6, and H13F

(A) Representation of the hydrophobic (green) and electrostatic (blue/red) surfaces of Hβ_2m and ΔN6 (lowest energy structures) using APBS (Baker et al., 2001) and Pymol (DeLano, 2002). Highlighted residues are Phe30, Pro32, Phe62, Leu64, Phe70 and His84 (sticks) that show some of the largest movements of sidechain orientation in the two structures (see also Figure 3C).

(B) ^{15}N longitudinal relaxation (R_1=1/T_1) and {^{1}H}^{15}N nOe relaxation measurements of 500 μM Hβ_2m (black) and 500 μM ΔN6 (red) at pH 7.5, 25 °C. Circles highlight residues for which no transverse relaxation rate could be determined due to resonance overlap, line broadening or the residue being a proline. Black crosses mark missing assignments. Rainbow coloured ribbons above indicate the secondary structure content of Hβ_2m and ΔN6 deduced from the final set of 30 lowest energy structures using DSSPcont (Carter et al., 2003). The error was estimated using duplicates.

(C) Cartoon overlay showing one monomer of the hexameric β_2m taken from the crystal structure of H13F (3CIQ, in blue) (Calabrese et al., 2008) and the lowest energy structure of ΔN6 (red). Pro32 (blue and red sticks, spheres, respectively) and the disulfide bond (Cys25-Cys80, sticks) are highlighted.

(D) RMS Cα [Å] of the overlay of the structures shown in (C). The rainbow coloured ribbon indicates the secondary structure elements of ΔN6 deduced from the final set of 30 lowest energy structures using DSSPcont (Carter et al., 2003).

(E) Overlay of the structures of H13F and ΔN6 showing residues whose sidechains differ most significantly (> 2 Å) in orientation in the two structures shown in (C).

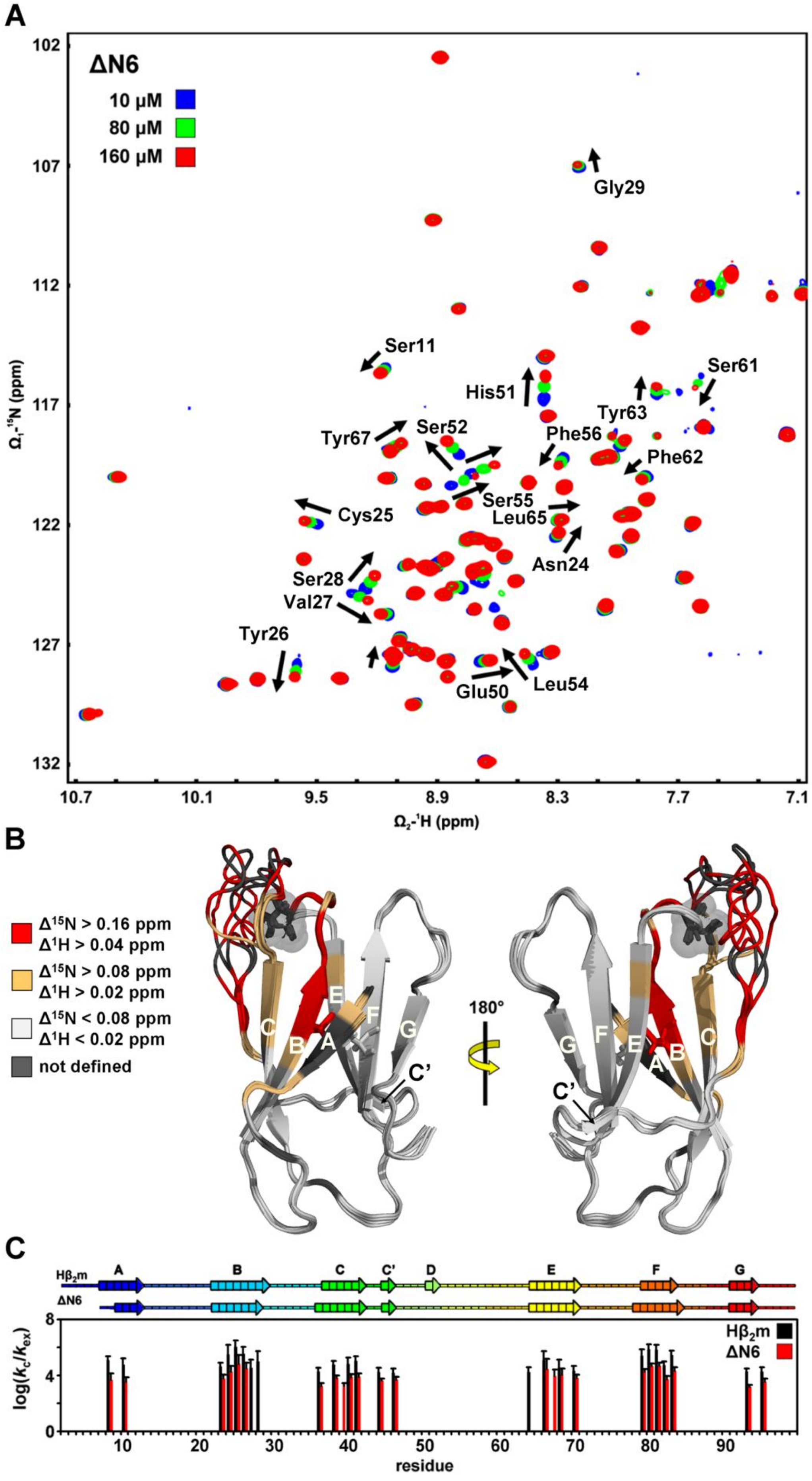
A
ΔN6
10 µM
80 µM
160 µM
Gly29
Ser11
His51
Ser61
Tyr63
Tyr67
Ser52
Phe56
Phe62
Ser55
Leu65
Cys25
Asn24
Ser28
Val27
Tyr26
Glu50
Leu54
Ω1-15N (ppm)
Ω2-1H (ppm)
B
Δ15N > 0.16 ppm Δ1H > 0.04 ppm
Δ15N > 0.08 ppm Δ1H > 0.02 ppm
Δ15N < 0.08 ppm Δ1H < 0.02 ppm
not defined
180°
C
Hβ2m
ΔN6
log(kc/kex)
residue

Figure S3, related to Figure 4. Hydrogen Exchange Kinetics of Native Hβ_2m and the Concentration Dependence of the ^{1}H-^{15}N Spectrum of ΔN6

(A) ^{1}H-^{15}N HSQC spectra of 10 μM (blue), 80 μM (green) and 160 μM (red) ΔN6 at pH 7.2, 25 °C. Resonances that shift significantly with protein concentration are labelled and highlighted with an arrow (pH 8.2 → pH 6.2).

(B) Cartoon representation of the five lowest energy structures of ΔN6. Highlighted in red and light orange are the residues that correspond to resonances that shift significantly (^{1}H / ^{15}N > 0.04 / 0.16 ppm or ^{1}H / ^{15}N > 0.02 / 0.08 ppm, respectively) shown in (A). Residues coloured in black are not assigned or are proline residues or, for residues in the A-strand, resonate in a crowded region in the spectrum and hence their chemical shift *versus* concentration could not be determined. Residues coloured in light grey do not show significant chemical shift (^{1}H / ^{15}N < 0.02 / 0.08 ppm) under the conditions employed.

(C) Hydrogen exchange kinetics of 80 μM Hβ_2m or ΔN6 measured at pH 7.2, 37 °C or pH 7.2, 25 °C, respectively. The intrinsic rates of HX (k_c) were calculated as described (Bai et al., 1993). Measured rates are denoted k_{ex}. The β-strands in native Hβ_2m and ΔN6 are shown above in rainbow colour. The rate of HX of ΔN6 is too fast to measure by NMR at 37 °C. The error was estimated from the noise level of the experiment.

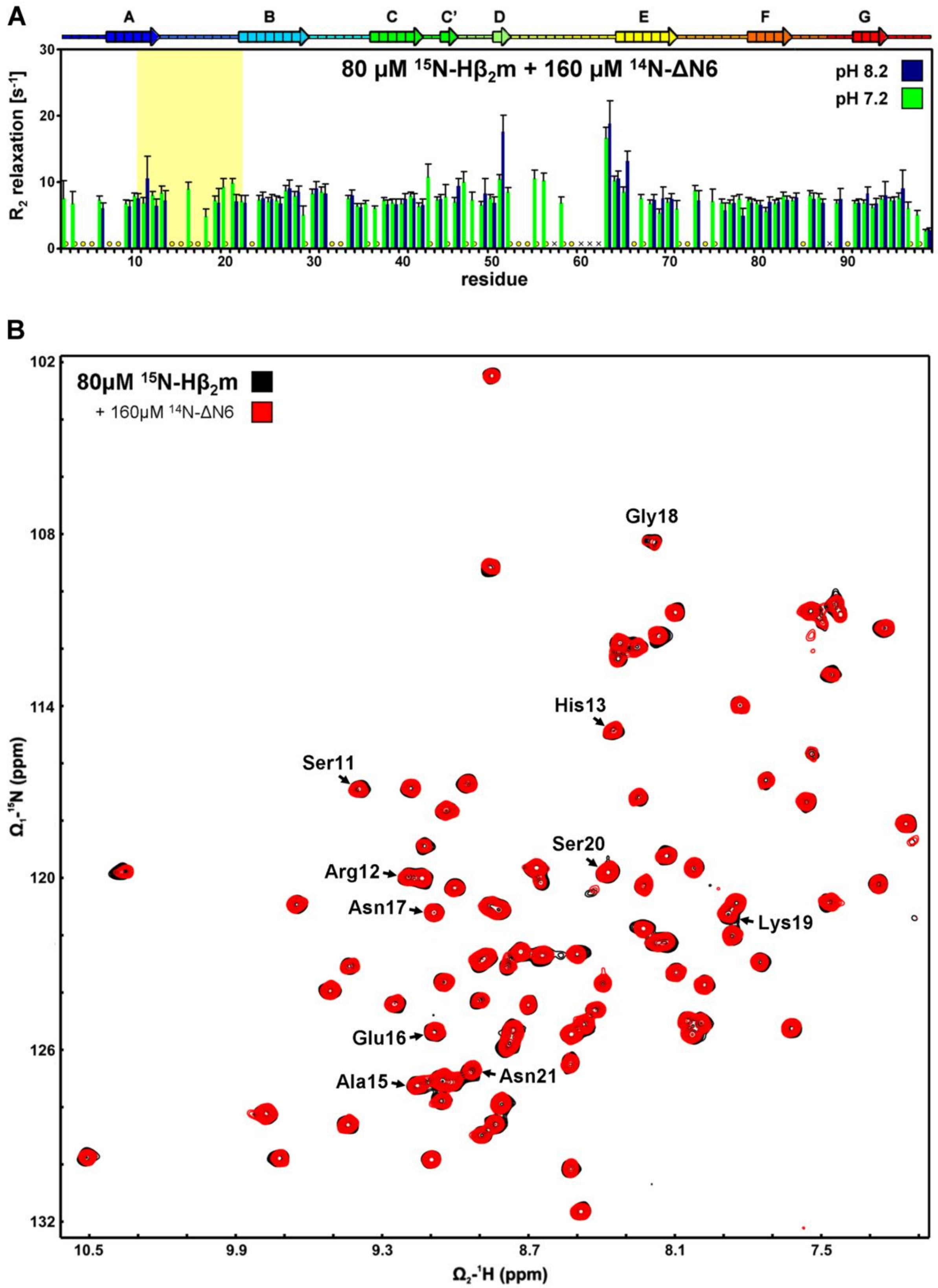

Figure S4, related to Figure 6. Interactions between Native Hβ_2m and ΔN6 Are pH Dependent

(A) ^{15}N transverse relaxation measurements (R_2=1/T_2) of 80 μM ^{15}N-Hβ_2m in the presence of 160 μM ^{14}N-ΔN6 at pH 8.2 or pH 7.2 (blue and green, respectively), 37 °C. Circles highlight residues for which no transverse relaxation rate could be determined due to resonance overlap, line broadening or the corresponding residue being a proline. Black crosses mark missing assignments. The rainbow coloured ribbon above indicates the secondary structure content of native Hβ_2m deduced from the final set of 30 lowest energy structures using DSSPcont (Carter et al., 2003). The error was estimated using duplicates.

(B) ^{1}H-^{15}N HSQC spectra of 80 μM ^{15}N-Hβ_2m alone (black) or presence of 160 μM ^{14}N-ΔN6 at pH 6.2 (red), 37 °C. Residues of Hβ_2m within the AB-loop (residues 11-21) that show enhanced transverse relaxation rates under those conditions (see Figure 6A) are highlighted.

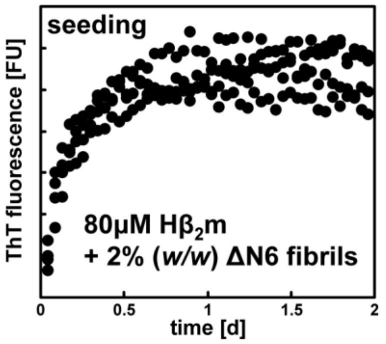

Figure S5, related to Figure 5. Cross-Seeding of Native Hβ_2m Using ΔN6 Fibrillar Seeds

ThT fluorescence of 80 µM Hβ_2m in 25 mM sodium phosphate buffer at pH 7.2, 37 °C seeded quiescently by using 2 % (*w/w*) ΔN6 fibrillar seeds grown *de novo* in 25 mM sodium phosphate buffer pH 7.2, 37 °C. Note: no fibrils form when 80 µM Hβ_2m is incubated without seed (Figure 5A).

Table S1, related to Figure 2. Assignment of *cis*- and *trans*-X-proline residues in native Hβ_2m and ΔN6

	Native Hβ_2m			ΔN6		
	^{13}Cβ [ppm]	^{13}Cγ [ppm]	Δ ^{13}Cβ^{13}Cγ [ppm]	^{13}Cβ [ppm]	^{13}Cγ [ppm]	Δ ^{13}Cβ^{13}Cγ [ppm]
Pro5	32.14 ± 0.14	26.99 ± 0.05	5.15 ± 0.19	-	-	-
Pro14	31.48 ± 0.08	28.21 ± 0.14	3.27 ± 0.22	31.44 ± 0.09	28.20 ± 0.09	3.24 ± 0.18
Pro32	35.08 ± 0.11	25.10 ± 0.13	9.98 ± 0.24	31.51 ± 0.12	27.17 ± 0.13	4.34 ± 0.25
Pro72	32.15 ± 0.07	26.82 ± 0.16	5.33 ± 0.23	32.15 ± 0.09	26.86 ± 0.08	5.29 ± 0.17
Pro90	31.97 ± 0.16	27.71 ± 0.14	4.26 ± 0.30	32.41 ± 0.09	27.17 ± 0.13	5.24 ± 0.22

^{13}Cβ and ^{13}Cγ chemical shifts of proline residues and their difference Δ^{13}Cβ^{13}Cγ in native Hβ_2m and ΔN6 are shown. Values of Δ^{13}Cβ^{13}Cγ < 4.80 ppm correspond to a 100 % probability that the proline is *trans*. Values > 9.15 ppm correspond to a 100 % probability that the proline is *cis*. Values between > 4.80 ppm and < 9.15 ppm indicate mixed probabilities (Schubert et al., 2002).

residue	construct	Total (i ± > 4) nOes	nOe	distance [Å]
Phe30	Native Hβ_2m	27	Ile35 HG → Phe30 HZ	3.0 ± 0.4
			Phe30 HB1 → Pro5 HA	3.0 ± 0.4
			Phe30 HA → Arg3 HB1	3.2 ± 0.5
			Leu64 HB1 → Phe30 HZ	3.3 ± 0.5
			Leu64 HD →Phe30 HZ	3.5 ± 0.5
	ΔN6	29	Phe30 HB2 → Ile35 HD	3.0 ± 0.5
			Phe30 HB1 → His84 HD	3.2 ± 0.6
			Phe30 HB1 → Ile35 HD	3.5 ± 0.5
			Val82 HG → Phe30 HZ	3.5 ± 0.6
			Ile35 HD → Phe30 HD	3.8 ± 0.5
Pro32	Native Hβ_2m	0		
	ΔN6	26	Pro32 HG1 → Leu64 HG	2.1 ± 0.3
			Pro32 HB2 → Leu64 HD	2.5 ± 0.5
			Leu64 HD → Pro32 HA	2.8 ± 0.4
			Leu64 HD → Pro32 HB2	3.4 ± 0.5
			Ile35 HG → Pro32 HA	3.6 ± 0.7
Ile35	Native Hβ_2m	33	Val27 HG → Ile35 HD	1.6 ± 0.3
			Ile35 HG → Leu64 HD	1.7 ± 0.3
			Ile35 HD → Val82 HG	2.1 ± 0.3
			His84 HD → Ile35 HG	2.4 ± 0.4
			Ile35 HD → Val82 HB	2.4 ± 0.5
	ΔN6	40	Ile35 HD → Val27 HG	1.6 ± 0.3
			Ile35 HD → Val82 HG	2.0 ± 0.4
			Ile35 HD → Leu64 HD	2.1 ± 0.5
			Ile35 HG → Val82 HG	2.8 ± 0.5
			Phe30 HB2 → Ile35 HD	3.0 ± 0.5
Phe62	Native Hβ_2m	6	Ser33 HB2 → Phe62 HD	4.4 ± 0.6
			Phe62 HD → Leu54 HD	5.3 ± 0.8
			Phe62 HD → Ser33 HB1	5.8 ± 0.6
			Phe56 HB2 → Phe62 HD	5.8 ± 0.7
			Phe56 HB1 → Phe62 HD	5.8 ± 0.7
	ΔN6	8	Phe62 HE → Pro32 HG2	4.9 ± 0.7
			Phe62 HZ → Pro32 HG2	5.1 ± 0.7
			Phe62 HE → Pro32 HG1	5.6 ± 0.9
			Phe62 HZ → Pro32 HB1	6.8 ± 0.8
			Pro32 HA → Phe62 HE	7.4 ± 1.0
Leu64	Native Hβ_2m	18	Ile35 HG → Leu64 HD	1.7 ± 0.3
			Leu64 HD → Asp34 HA	3.1 ± 0.4
			Leu64 HB1 → Phe30 HZ	3.3 ± 0.5
			Ile35 HN → Leu64 HD	3.5 ± 0.6
			Leu64 HD → Phe30 HZ	3.5 ± 0.5
	ΔN6	46	Ile35 HD → Leu64 HD	2.1 ± 0.5
			Pro32 HB2 → Leu64 HD	2.5 ± 0.5
			Leu64 HD → Pro32 HA	2.8 ± 0.4
			Ile35 HD → Leu64 HD	3.2 ± 0.6
			Val27 HB → Leu64 HD	3.3 ± 0.5
Phe70	Native Hβ_2m	27	Asn21 HN → Phe70 HN	3.0 ± 0.4
			Phe70 HN → Phe22 HA	3.4 ± 0.4
			Asn21 HB2 → Phe70 HD	3.5 ± 0.4
			Ser11 HB2 → Phe70 HZ	3.6 ± 0.6
			Leu23 HD → Phe70 HZ	3.7 ± 0.5
	ΔN6	36	Phe70 HN → Phe22 HA	3.6 ± 0.5
			Ser11 HB1 → Phe70 HZ	4.1 ± 0.8
			Asn21 HN → Phe70 HE	4.3 ± 0.5
			Leu23 HN → Phe70 HE	4.2 ± 0.5

			Phe78 HD → Phe70 HB1	4.5 ± 0.5
His84	Native Hβ_2m	21	His84 HD → Ile35 HG	2.4 ± 0.4
			Ile35 HA → His84 HD	3.0 ± 0.4
			Ile35 HB → His84 HD	3.2 ± 0.4
			His84 HN → Pro90 HA	3.3 ± 0.3
			Ile35 HG → His84 HD	3.3 ± 0.4
	ΔN6	18	Phe30 HB1 → His84 HD	3.2 ± 0.6
			Ile35 HA → His84 HA	3.3 ± 0.5
			His84 HD → Ile35 HB	3.7 ± 0.7
			Ile35 HG → His84 HB2	4.0 ± 0.5
			Ile35 HD → His84 HD	4.0 ± 0.6
Trp95	Native Hβ_2m	37	Ala15 HB2 → Trp95 HD	2.8 ± 0.4
			Val9 HG → Trp95 HB1	2.5 ± 0.4
			Tyr10 HA → Trp95 HD	2.8 ± 0.4
			Val9 HG → Trp95 HA	2.9 ± 0.3
			Leu23 HD → Trp95 HD	2.9 ± 0.5
	ΔN6	68	Val9 HG → Trp95 HA	2.8 ± 0.4
			Val9 HG → Trp95 HB1	2.8 ± 0.4
			Trp95 HN → Val9 HG	2.9 ± 0.3
			Leu23 HD → Trp95 HB1	3.2 ± 0.5
			Trp95 HE → Tyr10 HA	3.2 ± 0.4

Table S2, related to Figure 3. Five long-range (i ± > 4) nOes of native Hβ_2m and ΔN6 ranked for closest distance (pH 7.5, 25 °C) in the ensembles of structures calculated

The residues listed show the most intense long-range nOes for both constructs that define the arrangement of residues highlighted in Figure 3C. Note that a full annotation of chemical shifts and peak lists is deposited in BMRB with accession numbers 17165 and 17166 for Hβ_2m and ΔN6, respectively.

Supplemental Experimental Procedures

Assembly of amyloid fibrils. For all experiments a protein aliquot stored in 10 mM sodium phosphate buffer, pH 7.2 at -80 °C was thawed on ice and centrifuged (10 min, 10,000 *g*, 10 °C). For fibril growth assays samples (100 µl) were prepared in double sealed (UC-500 sealing film, Axygen) 96-well plates (Corning Incorporated, Costar) and contained 0.8-500 µM protein, 81-89.5 mM NaCl, 10 µM ThT and 0.02 % (*w/v*) sodium azide in 10 mM sodium phosphate buffer, pH 6.2-8.2. All reaction mixtures had an ionic strength of 100 mM and were sterile filtered (0.2 µm, Sarstedt). For seeded reactions, each sample contained additionally 10 % (*w/w*) ΔN6 seeds formed at pH 7.2, which were collected by centrifugation (5,000 *g*, 10 min) and resuspended in H_2O (3 times) prior to use. *De novo* fibril growth was carried out by incubating the plate at 37 °C with agitation (200 rpm) for several weeks. Approximately every 48 hours fibril growth was assessed by measuring the fluorescence of ThT (excitation 440 nm, emission 480 nm) using a Fluorostar Optima, BMG Labtech plate reader at 37 °C (40 readings per well, gain 1200). Seeded fibril growth was carried out by incubating the plate at 37 °C quiescently for a week. Every hour fibril growth was assessed by measuring the fluorescence of ThT (excitation 440 nm, emission 480 nm) using a Fluorostar Optima, BMG Labtech plate reader at 37 °C (40 readings per well, gain 1200). The soluble fraction obtained after centrifugation (14,000 *g*, 10 min) was analysed by SDS-PAGE.

Negative-stain EM. Carbon coated copper grids were prepared by the application of a thin layer of formvar with an overlay of thin carbon. Samples were centrifuged (14,000 g, 10 min) and the pellets resuspended in deionised water and then applied to the grid in a drop-wise fashion. The grid was then carefully dried with filter paper before it was negatively stained

by the addition of 18 µl of 2 % (*w/v*) uranyl acetate. Micrographs were recorded on a Philips CM10 electron microscope at moderate dose (~ 100 e $Å^{-1}$).

NMR spectroscopy and structure determination. Sequential assignments were obtained from analysis of HNCA, HNCO, HN(CO)CA, CBCA(CO)NH, HNHA and ^{1}H-^{15}N NOESY-HSQC (Vuister and Bax, 1993; Kay et al., 1994; Muhandiram and Kay, 1994; Zhang et al., 1994; Zhang et al., 1997). Spectra were processed using NMRPipe (Delaglio et al., 1995) and analysed in CCPN analysis (Vranken et al., 2005). Aliphatic sidechain resonances were assigned on the basis of H(C)CH-TOCSY, (H)CCH-TOCSY and (H)CCH-COSY (Bottomley et al., 1999). Aromatic specific sidechain assignments were made using ^{1}H-^{13}C CT HSQC, HB(CBCGCD)HD and HB(CBCGCDCE)HE spectra (Yamazaki et al., 1993) and short Hβ-Hδ nOes. For the measurement of residual dipolar couplings ^{1}H-^{15}N J-modulated HSQC spectra (Tjandra et al., 1996) were acquired in the presence and absence of 7 mg ml^{-1} or 15 mg ml^{-1} pf1 bacteriophage for $H\beta_2m$ and ΔN6, respectively. NOe distance restraints were derived from 120 ms three-dimensional ^{1}H-^{15}N NOESYHSQC, three-dimensional ^{1}H-^{13}C NOESY-HSQC (Muhandiram et al., 1993; Smallcombe et al., 1995) and three-dimensional aromatic ^{13}C filtered NOESY spectra. Torsion angles phi and psi were predicted from $^{1}H\alpha$, $^{13}C\alpha$, $^{13}C\beta$, C' and backbone ^{15}N chemical shifts using TALOS (Cornillescu et al., 1999). NOes were assigned and structures calculated in a two stage process. In the initial stage structures were calculated using the Marvin/PASD simulated annealing protocol (Kuszewski et al., 2004) from X-PLOR-NIH v2.17 (Schwieters et al., 2006) with all measured nOe peaks and TALOS restraints. In the second stage the 50 structures with the lowest energy were then transferred into the first round of an ARIA 2.1 calculation (Nilges et al., 1997) along with high probability assignments. The RDC alignment magnitude and rhombicity were calculated as an average from the initial 50 structures from the first stage

with the DC utility from NMRPipe (Delaglio et al., 1995) and RDCs were used as variable angle restraints (VEAN) and SANI restraints (Clore et al., 1998) with a force constant of 0.1. The final structures were refined in a water box using standard ARIA parameters, the length of the two slow cooling stages in the ARIA protocol were extended by a factor of four as described (Fossi et al., 2005). Network anchoring was switched on throughout the calculation (Linge et al., 2004). During refinement the nOe distance restraint network was corrected for spin diffusion. The adjustment is based on the calculation of a theoretical intensity matrix from the set of structures produced each iteration. The theoretical intensity values were then used to calibrate the experimental volumes, and to correct the distance target. The calibrated volumes could then be used to estimate the error. All ARIA calculations were carried out using CNS 1.1 (Brunger et al., 1998). The final structure ensemble of native Hβ_2m and ΔN6 was based on a total of 2065 or 2565 experimental nOe restraints, 128 or 118 dihedral angle restraints and 75 or 76 ^{1}H-^{15}N residual coupling restraints, respectively (Table 1, Table S2 and Figure 3). Structures were validated with WHAT-CHECK (Hooft et al., 1996) and PROCHECKNMR (Laskowski et al., 1996). The molecular structure figures were generated using MolMol (Koradi et al., 1996) and Pymol (DeLano, 2002).

SOFAST-HMQC NMR experiments were carried out as described (Schanda and Brutscher, 2005). For refolding experiments: Hβ_2m was denaturated in 8 M urea at 37 °C prior to refolding *via* 10-fold dilution with 25 mM sodium phosphate buffer pH 7.5 at 25 °C and the sample was then placed immediately into the NMR spectrometer. The first spectrum was recorded approximately 2 min after refolding was initiated. The length of each experiment was between 30 s and 5 min, d1 delays were between 0.500 and 0.550 seconds and the number of scans collected was between 2 and 8. Chemical shift referencing of all samples

was carried out using DSS as an internal standard for ^{1}H. ^{15}N and ^{13}C were indirectly referenced.

Secondary structure assignment: Using the continuous secondary structure assignment protocol DSSPcont (Carter et al., 2003), the secondary structure of native Hβ_2m and ΔN6 was calculated based on 30 lowest energy solution structures. The output suggests a likelihood for each residue to be in one of eight secondary structure states: 3_{10}-helix, pi-helix, helix-turn, extended beta sheet, beta bridge, bend, and other/loop. Each residue in native Hβ_2m and ΔN6 was individually assigned to the structure with the highest likelihood (usually > 50%) over all 30 members of the ensemble calculated. Accordingly, Hβ_2m contains eight β-strands (residues 6-11 (A), 21-28 (B), 36-41 (C), 44-45 (C'), 50-51 (D), 64-70 (E), 79-83 (F), 91-94 (G)). ΔN6 contains seven β-strands (residues 8-11 (A), 21-27 (B), 35-41 (C), 44-45 (C'), 64-70 (E), 78-84 (F), 91-94 (G)), while residues 50 and 51 that form β-strand D in native Hβ_2m adopted variable conformations in the 30 lowest energy structures calculated for ΔN6 with only 32 % and 0 % of molecules adopting β-strand conformation for these residues, respectively. By contrast in native Hβ_2m residues 50 and 51 establish both 53 % β-strand structure. β-strand C' in native Hβ_2m has a likelihood of 100 % whereas the likelihood in ΔN6 is reduced to 74 %. The 3_{10}-helix for residues 32-34 probably stabilises the *trans*-isomer of X-Pro32 and has a likelihood of 100 % in ΔN6 whereas the likelihood in native Hβ_2m is 0 %.

^{15}N NMR relaxation experiments. Backbone ^{15}N transverse relaxation (R_2=1/T_2), ^{15}N longitudinal relaxation (R_1=1/T_1), {^{1}H}^{15}N nOe relaxation measurements were carried out as described (Farrow et al., 1994). Duplicate measurements and spectral noise levels were used to obtain an estimate of the error. The R_2 relaxation measurements of all constructs were

performed at 500 MHz using a series of 10 experiments with relaxation delays ranging from 16.512 ms to 165.12 ms. The R_1 relaxation measurements of all constructs were performed at 500 MHz using a series of 11 experiments with mixing times ranging from 0 s to 1.28 s. For $\{^1H\}^{15}N$ nOe relaxation time experiments amide protons were pre-saturated with 120 ° pulses for 3.5 s prior to the experiment. All relaxation measurements were performed using 80-500 µM protein in 81-89.5 mM NaCl (giving a total ionic strength of 100 mM), 10 % (*v/v*) D_2O and 0.02 % (*w/v*) sodium azide in 10 mM sodium phosphate buffer, pH 6.2-8.2.

Hydrogen Exchange NMR. All samples were adjusted to pH 7.2 or 6.2 in 10 mM sodium phosphate buffer and freeze-dried prior to dissolving them in 85 or 89.5 mM NaCl (to a total ionic strength of 100 mM), 100 % (*v/v*) D_2O and 0.02 % (*w/v*) sodium azide. The dead-time of the experiment was approximately 5-10 min and data acquisition (5-15 min) was carried out using SOFAST-HMQC NMR methods (Schanda et al., 2005). The data obtained were fitted (Origin, Originlab©) to a single exponential and the error was estimated from the noise level of the experiment.

Supplemental References

Bai, Y., Milne, J.S., Mayne, L., and Englander, S.W. (1993). Primary structure effects on peptide group hydrogen exchange. Proteins *17*, 75-86.

Baker, N.A., Sept, D., Joseph, S., Holst, M.J., and McCammon, J.A. (2001). Electrostatics of nanosystems: application to microtubules and the ribosome. Proc Natl Acad Sci USA *98*, 10037-10041.

Bottomley, M.J., Macias, M.J., Liu, Z., and Sattler, M. (1999). A novel NMR experiment for the sequential assignment of proline residues and proline stretches in 13C/15N-labeled proteins. J Biomol NMR *13*, 381-385.

Brunger, A.T., Adams, P.D., Clore, G.M., DeLano, W.L., Gros, P., Grosse-Kunstleve, R.W., Jiang, J.S., Kuszewski, J., Nilges, M., Pannu, N.S., *et al.* (1998). Crystallography & NMR system: A new software suite for macromolecular structure determination. Acta Crystallogr D Biol Crystallogr *54*, 905-921.

Clore, G.M., Gronenborn, A.M., and Tjandra, N. (1998). Direct structure refinement against residual dipolar couplings in the presence of rhombicity of unknown magnitude. J Magn Reson *131*, 159-162.

Cornillescu, G., Delaglio, F., and Bax, A. (1999). Protein backbone angle restraints from searching a database for chemical shift and sequence homology. J Biomol NMR *13*, 289-302.

Delaglio, F., Grzesiek, S., Vuister, G.W., Zhu, G., Pfeifer, J., and Bax, A. (1995). NMRPipe: a multidimensional spectral processing system based on UNIX pipes. J Biomol NMR *6*, 277-293.

Fossi, M., Oschkinat, H., Nilges, M., and Ball, L.J. (2005). Quantitative study of the effects of chemical shift tolerances and rates of SA cooling on structure calculation from automatically assigned NOE data. J Magn Reson *175*, 92-102.

Hooft, R.W., Vriend, G., Sander, C., and Abola, E.E. (1996). Errors in protein structures. Nature *381*, 272.

Kay, L.E., Xu, G.Y., and Yamazaki, T. (1994). Enhanced sensitivity triple resonance spectroscopy with minimal H2O saturation. J Magn Res *A 109*, 129-133.

Koradi, R., Billeter, M., and Wuthrich, K. (1996). MOLMOL: a program for display and analysis of macromolecular structures. J Mol Graph *14*, 51-55, 29-32.

Laskowski, R.A., Rullmannn, J.A., MacArthur, M.W., Kaptein, R., and Thornton, J.M. (1996). AQUA and PROCHECK-NMR: programs for checking the quality of protein structures solved by NMR. J Biomol NMR *8*, 477-486.

Linge, J.P., Habeck, M., Rieping, W., and Nilges, M. (2004). Correction of spin diffusion during iterative automated NOE assignment. J Magn Reson *167*, 334-342.

Muhandiram, D.R., Farrow, N.A., Xu, G.Y., and Kay, L.E. (1993). A gradient ^{13}C NOESY-HSQC experiment for recording NOESY spectra of ^{13}C-labelled proteins dissolved in H_2O. J Magn Res *B 102*, 317-321.

Muhandiram, D.R., and Kay, L.E. (1994). Gradient enhanced triple resonance three dimensional NMR experiment with improved sensitivity. J Magn Res *B 103*, 203-216.

Nilges, M., Macias, M.J., O'Donoghue, S.I., and Oschkinat, H. (1997). Automated NOESY interpretation with ambiguous distance restraints: the refined NMR solution structure of the pleckstrin homology domain from beta-spectrin. J Mol Biol *269*, 408-422.

Schanda, P., Kupce, E., and Brutscher, B. (2005). SOFAST-HMQC experiments for recording two-dimensional heteronuclear correlation spectra of proteins within a few seconds. J Biomol NMR *33*, 199-211.

Schubert, M., Labudde, D., Oschkinat, H., and Schmieder, P. (2002). A software tool for the prediction of Xaa-Pro peptide bond conformations in proteins based on 13C chemical shift statistics. J Biomol NMR *24*, 149-154.

Schwieters, C.D., Kuszewski, J., and Clore, G.M. (2006). Using Xplor-NIH for NMR molecular structure determination. Prog Nuc Magn Res Spec, 47-62.

Smallcombe, S.H., Patt, S.L., and Keifer, P.A. (1995). WET solvent suppression and its applications to LC NMR and high resolution NMR spectroscopy. J Magn Res *A 117*, 295-303.

Tjandra, M., Grzesiek, S., and Bax, A. (1996). Magnetic field dependence of nitrogen-proton J splittings in ^{15}N enriched human ubiquitin resulting from relaxation interference and residual dipolar coupling. J Am Chem Soc, 6264-6272.

Vranken, W.F., Boucher, W., Stevens, T.J., Fogh, R.H., Pajon, A., Llinas, M., Ulrich, E.L., Markley, J.L., Ionides, J., and Laue, E.D. (2005). The CCPN data model for NMR spectroscopy: development of a software pipeline. Proteins *59*, 687-696.

Vuister, G.W., and Bax, A. (1993). Quantitative J correlation: A new approach for measuring homonuclear three bond J(HNHalfa) coupling constants in 15N enriched proteins. J Am Chem Soc *115*, 7772-7777.

Yamazaki, T., Forman-Kay, J.D., and Kay, L.E. (1993). Two dimensional NMR experiment for correlating ^{13}Cβ and ^{1}Hδ/ε chemical shifts of aromatic residues in 13C labeled proteins via scalar couplings. J Am Chem Soc, 11054-11055.

Zhang, O., Kay, L.E., Olivier, J.P., and Forman-Kay, J.D. (1994). Backbone 1H and 15N resonance assignments of the N-terminal SH3 domain of drk in folded and unfolded states using enhanced-sensitivity pulsed field gradient NMR techniques. J Biomol NMR *4*, 845-858.

Zhang, W., Smithgall, T.E., and Gmeiner, W.H. (1997). Three-dimensional structure of the Hck SH2 domain in solution. J Biomol NMR *10*, 263-272.
